# Identification and validation of roles of lysyl oxidases in the predictions of prognosis, chemotherapy and immunotherapy in glioma

**DOI:** 10.3389/fphar.2022.990461

**Published:** 2022-08-31

**Authors:** Qin-Xuan Xia, Jing Yu, Zhao-Jun Wang, Qi-Wen Guan, Xiao-Yuan Mao

**Affiliations:** ^1^ Department of Clinical Pharmacology, Xiangya Hospital, Central South University, Changsha, China; ^2^ Hunan Key Laboratory of Pharmacogenetics, Institute of Clinical Pharmacology, Central South University, Changsha, China; ^3^ Engineering Research Center of Applied Technology of Pharmacogenomics, Ministry of Education, Changsha, China; ^4^ National Clinical Research Center for Geriatric Disorders, Xiangya Hospital, Central South University, Changsha, Hunan, China

**Keywords:** lysyl oxidases, glioma, prognosis, chemotheraperapy, immunotherapy

## Abstract

**Background**: Previous investigations have illustrated that lysyl oxidase family enzymes (LOXs) are contributing factors for tumor progression and remodeling immunomicroenvironment. However, it is scarce regarding comprehensive analysis of LOXs in the predictions of prognosis, chemotherapy and immunotherapy in glioma, the highly invasive brain tumor. Our present work aimed to explore the prognostic value, chemotherapeutic drug sensitivity and immunotherapy according to distinct LOXs expressions in glioma through bioinformatics analysis and experimental verification.

**Methods:** We collected gene expression data and clinical characteristics from the public databases including Chinese Glioma Genome Atlas (CGGA)-325, CGGA-693, the Cancer Genome Atlas (TCGA), IMvigor210 and Van Allen 2015 cohorts. The correlations between the clinicopathological factors and differential LOXs expressions were analyzed. The ROC curve and Kaplan-Meier analysis were conducted to evaluate the prediction ability of prognosis. Chemotherapeutic drug sensitivity *via* distinct LOXs expression levels was predicted using the pRRophetic package. Immune score, immune cell infiltration and immune checkpoint expression levels were also analyzed through diverse algorithms in R software. Finally, mRNA and protein expressions of LOXs were validated in glioma cells (T98G and A172) by real-time quantitative PCR and Western blot, respectively.

**Results:** Our results demonstrated that high levels of LOXs expressions were positively associated with glioma grades, older age and MGMT unmethylated status while elevations of LOXs were negatively correlated with IDH mutation or 1p/19q co-deletion. Furthermore, the glioma patients with low levels of LOXs also exhibited better prognosis. Also, differential LOXs expressions were associated with at least 12 chemotherapeutic drug sensitivity. Besides, it was also found that glioma patients with high LOXs expressions showed higher enrichment scores for immune cell infiltration and increased levels of immune checkpoints, suggesting the critical role of distinct LOXs expression levels for glioma immunotherapy. The predictive roles of LOXs expression in tumor immunotherapy were also validated in two immunotherapy cohorts including IMvigor 210 and Van Allen 2015. Experimental results revealed that expressions of LOX, LOXL1, LOXL2, and LOXL3 were higher in glioma cell lines at mRNA and protein levels.

**Conclusion:** Our findings altogether indicate that LOXs have potent predictive value for prognosis, chemotherapy and immunotherapy in glioma patients.

## Graphical Abstract

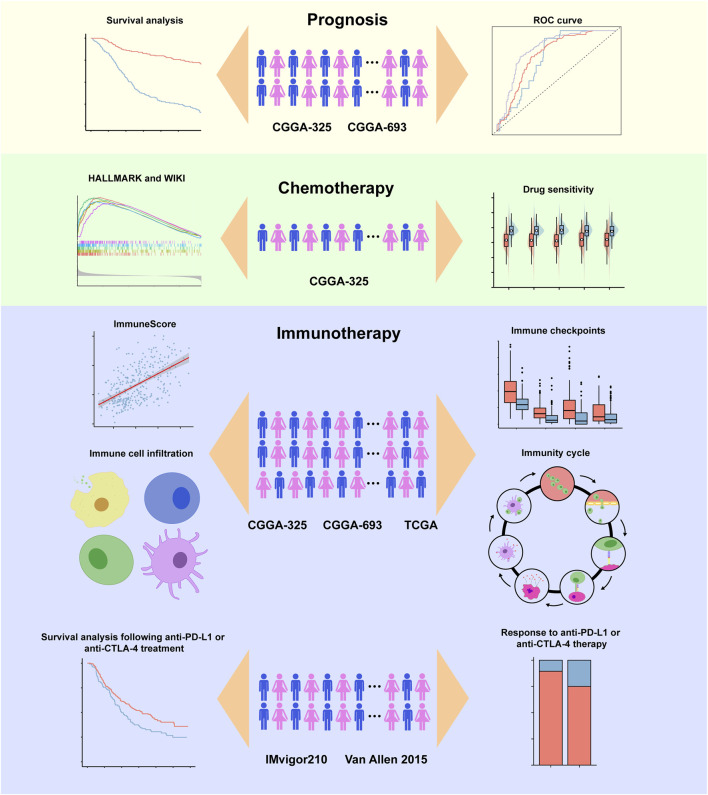

## Introduction

Glioma accounts for nearly 70% of malignant primary brain tumors in adults, which has high rate of tumor recurrence and poor clinical outcome (Ostrom et al., 2017). Generally, there are four grades of glioma, among which grade II and III are defined as diffuse lower-grade gliomas (LGG) whereas grade IV is regarded as high-grade glioma (HGG), also namely glioblastoma (GBM) (Brat et al., 2015). The overall survival rate is quite low in all of different grades of gliomas. In detail, the median survival time (MST) is only 16 months for GBM (Gilbert et al., 2014) while the MST for LGG patients ranges from 1 to 15 years (Lapointe et al., 2018). Although great progress has been made in surgical resection, radio- and chemotherapy, the glioma patients are still succumbed to tumor recurrence and malignant progression. One of the major reasons is possibly a result of the diffuse infiltration of cancer cells into the brain parenchyma (Louis, 2006), which is unlikely to implement the complete surgical resection. Despite mutations in isocitrate dehydrogenase (IDH) and co-deletion of the short arm of chromosome 1 and the long arm of chromosome 19 (1p/19q) have been identified as critical indicators for disease diagnosis, treatment option and prognostic evaluation (Wick et al., 2014; Lee et al., 2018), targeting these molecular markers still has minimal effects on glioma patients. Therefore, it is of urgent need to explore novel biomarkers to predict the prognosis and response to therapies in glioma.

Tumor microenvironment (TME), developed from the concept of “seed and soil” by Paget in the 1880s (Paget, 1889), have emerged as a promising therapeutic target for cancer treatment in recent years due to the vital roles of TME in the regulations of cancer progression and therapeutic outcome (Bejarano et al., 2021). The TME typically comprises stromal cells (e.g., immune cells and fibroblasts) and the extracellular matrix (ECM) as well as other secreted molecules such as growth factors, cytokines and chemokines (Bejarano et al., 2021). ECM remains the critical components in TME which plays a crucial role in the interaction between cancer cell and stromal cell. The importance of the surrounding ECM in the acceleration of glioma progression is widely appreciated due to the contribution of ECM to the cellular transformation and metastasis (Haj-Shomaly et al., 2022). Furthermore, the ECM protein has also been reported to reprogram infiltrating immune cells in glioma microenvironment (Broekman et al., 2018; Kiyokawa et al., 2021), finally influencing the glioma phenotype.

Lysyl oxidase family enzymes (LOXs) including LOX and LOX-like (LOXL) 1-4 are key ECM-modifying monoamine oxidases, which catalyze the cross-linking of ECM proteins such as collagen and elastin, thereby facilitating the stability of ECM (Cox and Erler, 2011). Recently, the roles of LOXs in tumor formation and progression have been extensively reported. For example, up-regulation of LOX is previously reported to trigger excessive collagen deposition and subsequently enhance cancer cell proliferation and invasiveness in *Lkb1*-deficient lung tumors (Gao et al., 2010). Besides, high LOX expression in the cancer-associated fibroblasts can also remodel the matrix collagen microenvironment and increase matrix stiffness and finally promote oral squamous cell carcinoma progression (Zhang et al., 2021). In glioma research, the increase of LOX protein expression is positively associated with the malignant grade of astrocytomas and GBM with IDH1 mutation always exhibits lower LOX expression levels (da Silva et al., 2015) compared with the wild type group. Furthermore, LOX is also identified as a potent macrophage chemoattractant *via* activating β1 integrin-dependent PYK2 signaling, finally promoting glioma progression (Chen et al., 2019). Regarding the roles of LOXL1-4 in the glioma, LOXL1 is also demonstrated to exhibit antiapoptotic activity (Yu et al., 2020) and promote glioma cell proliferation (Li et al., 2018) while the facilitation of cell invasion for LOXL3 is observed in glioma cell line U87MG (Laurentino et al., 2021). However, a comprehensive analysis of the roles of LOXs in the glioma biology and the therapeutic response has not been elucidated.

Herein, our present study was performed to comprehensively evaluate the roles of LOXs expressions in the prognosis, chemotherapy, and immunotherapy in glioma patients in the public databases including Chinese Glioma Genome Atlas (CGGA)-693, CGGA-325 and The Cancer Genome Atlas (TCGA). The validation of the roles of LOXs in tumor immunotherapy was also conducted in other cohorts including IMvigor210 and Van Allen 2015 datasets. Additionally, experimental results also revealed that expressions of LOX, LOXL1, LOXL2, and LOXL3 were higher in glioma cell lines (T98G and A172) at mRNA and protein levels compared with the normal astrocyte HEB. It was concluded that distinct LOXs expressions have significant contributions to the predictions of prognosis, chemotherapy and immunotherapy in glioma.

## Materials and methods

### Data source and processing

RNA-seq data and clinical information of TCGA database were downloaded from the UCSC Xena platform (https://xenabrowser.net/datapages/). CGGA-325 and CGGA-693 with detailed transcriptomic data and clinical annotations were different datasets in the CGGA platform and from CGGA website (http://www.cgga.org.cn/). Two clinical cohorts including IMvigor210 and Van Allen 2015 which were selected for the validation of roles of LOXs in tumor immunotherapy were obtained from the “IMvigor210CoreBiologies” package (http://research-pub.gene.com/IMvigor210Core.

Biologies) and cBioPortal database (http://www.cbioportal.org/), respectively. After data filtration, the samples without survival information in the selected datasets were excluded. Among these datasets, CGGA-325 was selected as the training set while CGGA-693, TCGA, IMvigor210, and Van Allen 2015 cohorts were chosen for validation. The purpose of data analysis and the corresponding package were summarized in Supplementary Figure S1. The detailed clinicopathological characteristics of glioma patients were summarized in Supplementary Table S1. The raw data from CGGA-325, CGGA-693 or TCGA were downloaded in the form of fragments per kilobase of transcript per million reads sequenced (FPKM) and transformed to transcripts per kilobase million (TPM). The basic information of two cohorts including IMvigor 210 and Van Allen 2015, which was chosen for the verification of the roles of LOXs in tumor immunotherapy, was summarized in Supplementary Table S2. Characteristics of the five datasets selected in our present work and the number of patients with survival information were listed in Supplementary Table S3.

### Analysis of mRNA expressions of LOXs in glioma

The correlation of LOXs expression levels and World Health Organization (WHO) grades, IDH mutation or 1p/19q co-deletion were analyzed *via* Kruskal–Wallis test for the comparisons among more than three groups and Mann-Whitney *U* test for the comparison between two groups and visualized by “ggplot2” package (Villanueva and Chen, 2019) of R software with *p* value less than 0.05 considering to be statistically significant.

### Independent prognostic analysis

The “surv_cutpoint” function of “survminer” package (http://www.sthda.com/english/rpkgs/survminer/) was employed to determine the optimal cut-off value as previously described (Yu et al., 2020). Using this method, high and low LOXs expression profiles were divided, which was then selected for prognosis prediction *via* Kaplan-Meier (KM) survival analysis. The statistical significance was defined as the log rank test *via* “survival” package (https://CRANR-projectorg/package=survival). Further, receiver operating characteristic (ROC) curve was prepared to analyze predictive performance of 1, 3 or 5 years with differential LOXs expressions by calculating areas under curve (AUC) using “timeROC” package (Blanche et al., 2013).

Cox proportional hazards regression and meta-analysis *via* differential LOXs expressions were conducted using the “coxph” function in the “survival” package (https://CRANR-projectorg/package=survival) and the “metagen” function in the “meta” packages (http://CRAN.R-project.org/package=meta), respectively.

### Functional annotation and pathway enrichment analysis

Gene Set Enrichment Analysis (GSEA) (Subramanian et al., 2005) is a ubiquitously used tool to assess whether a given gene set shows statistical significance between two biological states. In the CCGA-325 dataset, 309 samples in this dataset were classified into two groups including high LOXs expression and low LOXs expression group. After that, the indicated gene sets from Molecular Signatures Database MSigDB version 7.5.1 (http://software.broadinstitute.org/gsea/msigdb) for H (HALLMARK pathway gene sets) (Liberzon et al., 2015) and C2 (WIKI pathways gene sets) (Kutmon et al., 2016) were used for GSEA analysis as previously described (Duran-Frigola et al., 2020; Cao et al., 2022). Enrichment score (ES) for all the gene sets shown above were calculated by randomizing the gene labels, and thus attributed values. The normalization of enrichment scores (NES) was then conducted by normalizing the ES for each gene set to account for the size of the set. *p* value and false discovery rate (FDR) q-values were used for evaluation of statistical significance. GSEA was conducted using “ClusterProfiler” package (version 4.2.2) of R software (Yu et al., 2012) and the GSEA enrichment plots were generated by “enrichplot” package of R software (https://github.com/GuangchuangYu/enrichplot).

### Prediction of the potential chemotherapeutic agents

The response to chemotherapeutic drugs was estimated through the Genomics of Drug Sensitivity in Cancer (GDSC) database (https://www.cancerrxgene.org/) according to the previous investigation (Jaaks et al., 2022). The prediction of the half-maximal inhibitory concentration (IC50) in the high LOXs and low LOXs expression groups was conducted *via* the “pRRophetic” package of R software (Geeleher et al., 2014).

### Depiction of tumor immune microenvironment in glioma

Immune score for each sample in the datasets selected in our present study (CGGA-325, CGGA-693, and TCGA cohorts) were evaluated using the “estimate” package of R software (Yoshihara et al., 2013) as previously described (Hong et al., 2022). Spearman correlation analysis was employed to analyze the relationship between differential LOXs expressions and immune score. The characteristics of TIME include infiltrations of immune cells, activation of anti-cancer immunity cycle and expressions of immune checkpoints (Chen and Mellman, 2013; Gajewski et al., 2013; Binnewies et al., 2018; DePeaux and Delgoffe, 2021). In this study, 667 immunomodulators were obtained from immune cells referred to Charoentong laboratory (Charoentong et al., 2017) using ssGSEA (single sample gene set enrichment analysis) algorithm belong to “gene set variation analysis (GSVA)” package of R software (Hänzelmann et al., 2013). Prior work has shown that there are seven steps involving in the activation of anti-cancer immunity cycle from Tumor Immunophenotype (TIP) database (http://biocc.hrbmu.edu.cn/TIP/), which includes seven steps as follows: step 1, antigen release by cancer cells; step 2, cancer antigen processing and presentation; step 3, T cell initiation and activation; step 4, T cells trafficking to cancer lesions; step 5, T cells infiltration into tumors; step 6, recognition of cancer cells by T cell and step 7, killing of cancer cells. These steps could be scored by ssGSEA based on gene expression of each sample. The score of each step reflected the activation of anti-tumor immunity. We then analyzed the expression levels of four well-known immune checkpoints including PD-L1, PD-1, CTLA-4, and IDO-1 (Xu et al., 2020) and they were regarded as key targets for glioma immunotherapy.

### Cell culture

HEB cell, a normal human brain astrocyte cell line, was provided from BeNa Culture Collection (China) and glioma cell lines T98G and A172 from American Type Culture Collection (United States) were cultured in Dulbecco’s modified Eagle’s medium with high glucose supplemented with 10% fetal bovine serum (FBS) and 1% penicillin/streptomycin in an incubator with 5% CO_2_ atmosphere at 37°C.

### Real-time quantitative polymerase chain reaction

Total RNA of cell samples from different groups was extracted using ice-cold Trizol reagent (Invitrogen). RNA purity was assessed using a Nanodrop One spectrophotometer (Thermo Fisher Scientific, United States) with the wavelength at 260/280 nm. One μg of total RNA was employed for the synthesis of cDNA using the SYBR Perfect real-time series kits (TaKaRa). After that, RT-qPCR was conducted on a LightCycler 480 machine (Roche). The complete reactions were shown as follows: 40 cycles of 10 s at 95°C and 20 s at 60°C. Expression of each target gene was calculated using the 2^−ΔΔCt^ method. The detailed information of primer sequences used in our present work was summarized in Supplementary Table S4.

### Western blot

Cell samples were collected and lysed by homogenization in ice-cold lysis buffer (P0013, Beyotime Biotechnology Institute, China) supplemented with protease inhibitor. After centrifugation at 13,000 *g* for 20 min, the supernatants were obtained and mixed with loading buffer and then boiled for 5 min. Equal amount of protein (20 μg) from different groups was loaded on gels for electrophoresis and membrane transferring. Afterwards, the membranes were blocked with 5% non-fat milk for 1 h and subsequently rinsed with primary antibodies including LOX (ab174316, rabbit, 1:5,000, 47 KDa), LOXL1 (ab81488, rabbit, 1:500, 63 KDa), LOXL2 (ab179810, rabbit, 1:500, 87 KDa), LOXL3 (ab232878, rabbit, 1:1,500, 28 KDa) and LOXL4 (ab130646, rabbit, 1:300, 84 KDa) and α-tubulin (AF0001, rabbit, 1:1,000, 50 KDa) were used as control. The next day, following several washes with Tris-buffered saline with Tween-20 (TBST), the membranes were then incubated with goat-anti-rabbit IgG secondary antibody for approximately 1 h at room temperature. The bands were observed under an enhanced chemiluminescence system (ChemiDox XRS; Bio-Rad, Hercules, CA, United States). The grayscale values for each protein were quantified using ImageJ software (NIH, Bethesda, MD, United States).

### Statistical analysis

For bioinformatics data analysis, all statistical data were analyzed using R software (version 4.1.3). Kruskal–Wallis test was used for the comparisons among more than three groups while Mann-Whitney *U* test was applied for the comparison between two groups. Correlation analysis between variables was evaluated using Spearman’s rank correlation coefficient. The overall survival (OS) of the glioma patients between different groups was analyzed using KM curves with the log-rank test. Univariate Cox regression model were employed for calculating hazard ratios (HRs).

For experimental data analysis, one-way ANOVA analysis was carried out to assess statistical significance among different groups. A *p* value less than 0.05 was considered as the statistical significance.

## Results

**FIGURE 1:**
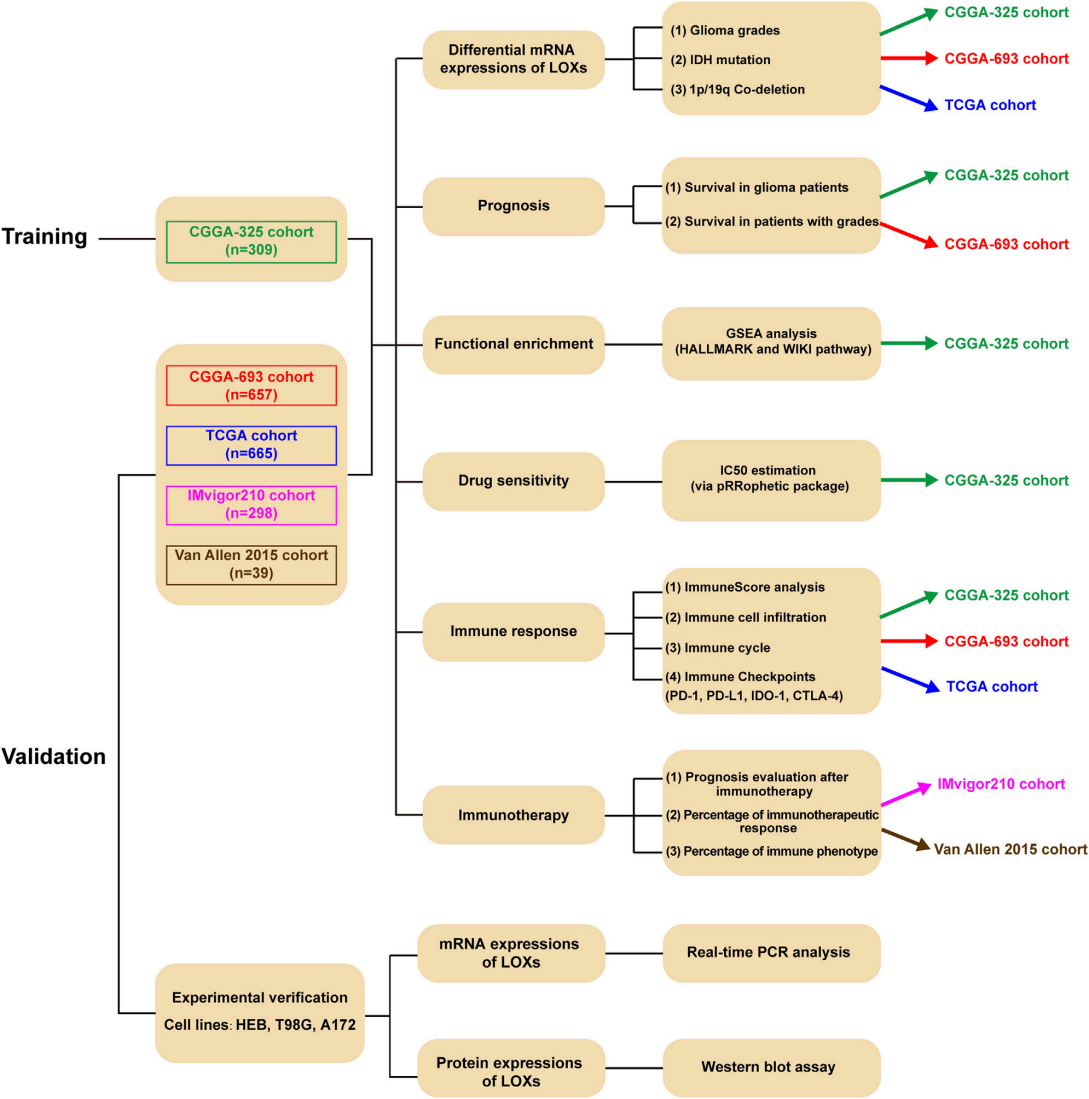
The workflow chart of our present study.

The workflow diagram of the present study was indicated in Figure 1. In the training stage, the RNA sequencing data were obtained from CGGA-325 dataset including 309 glioma samples while these data from CGGA-693 (657 samples), TCGA (665 samples), IMvigor 210 (298 samples) and Van Allen 2015 (39 samples) were chosen as validation datasets. Experimental verification was further carried out in various glioma cell lines including T98G and A172 and human normal astrocyte HEB with respect to mRNA and protein expressions of LOXs. Throughout the bioinformatics analysis, all the samples used in the present work have the complete survival information.

### Relationship between LOXs expressions and clinicopathological features in glioma

**FIGURE 2:**
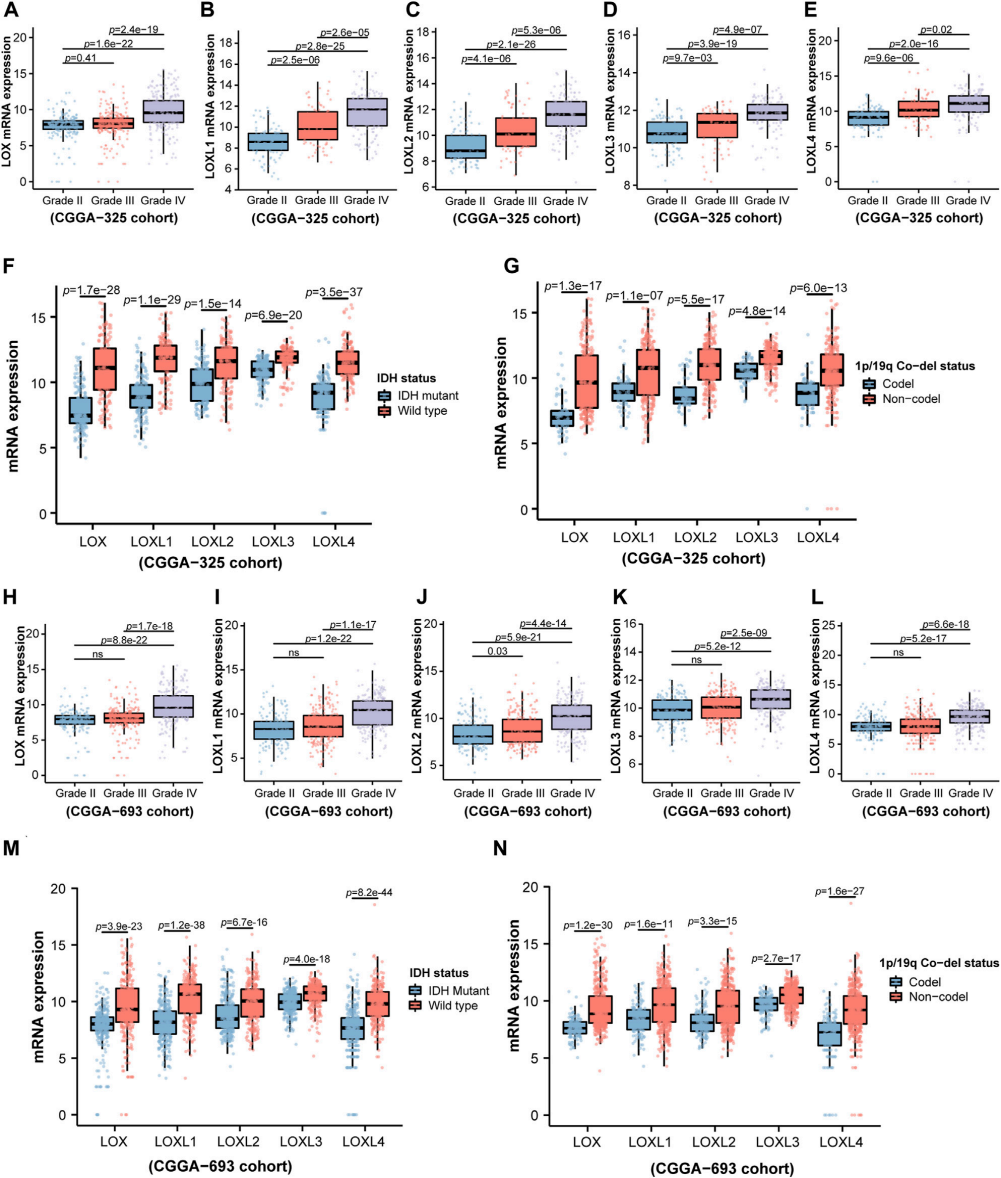
Analysis of relationship between LOXs expressions and WHO grade, IDH mutation status or 1p/19q co-deletion status in glioma in CGGA-325 and CGGA-693 cohorts. **(A–E)** Indicates mRNA expression levels of LOXs including LOX and LOXL1-4 in different glioma grades according to WHO in CGGA-325; **(F)** mRNA expressions of LOXs in IDH-mutation and wild type groups in CGGA-325; **(G)** mRNA expressions of LOXs in 1p/19q co-deletion and 1p/19q non-co-deletion groups in CGGA-325; **(H–L)** validation of the mRNA expression levels of LOXs in different glioma grades according to WHO in CGGA-693; **(M)** validation of mRNA expressions of LOXs in IDH-mutation and wild type groups in CGGA-693; **(N)** validation of mRNA expressions of LOXs in 1p/19q co-deletion and 1p/19q non-co-deletion groups in CGGA-693.

**FIGURE 3:**
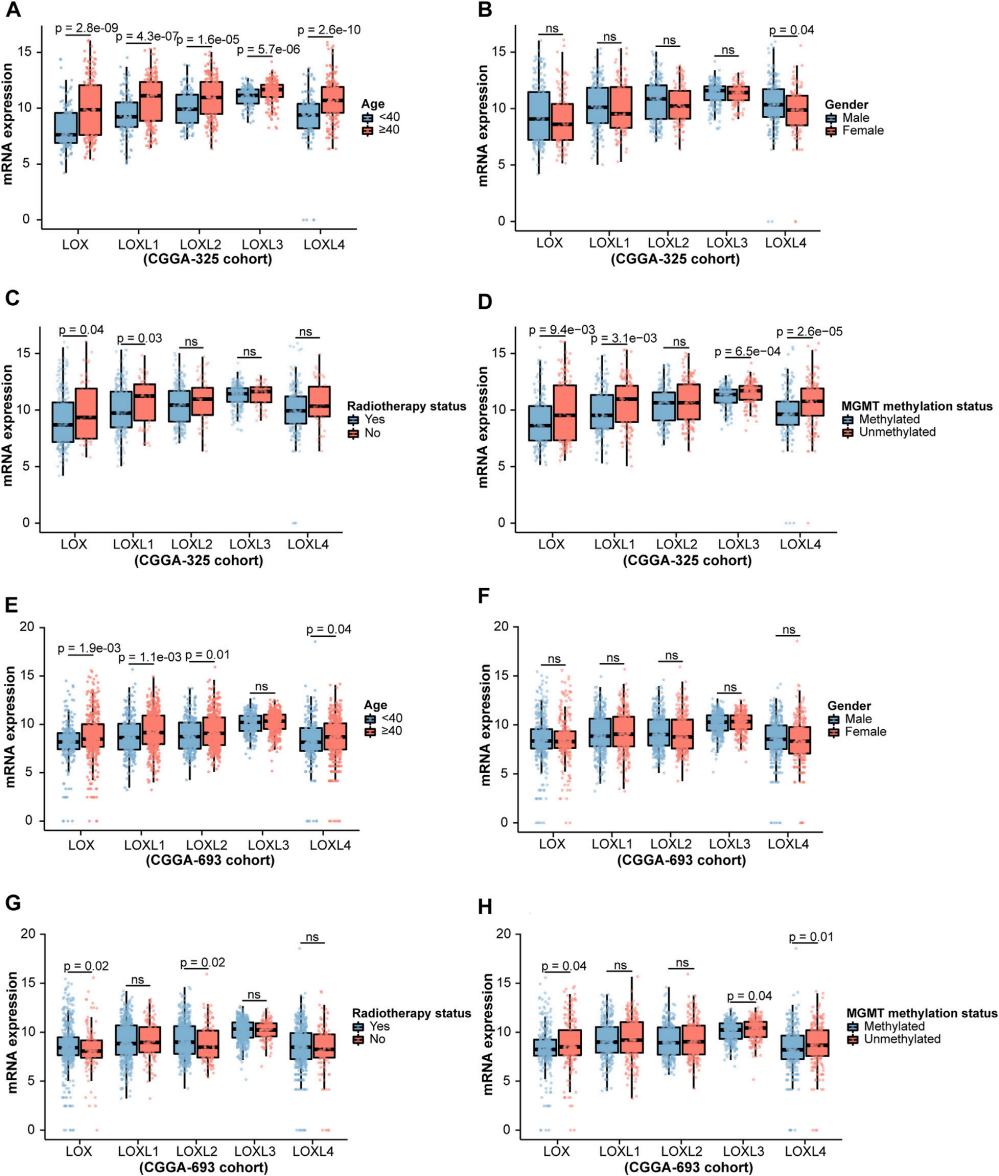
Analysis of relationship between LOXs expressions and age, gender, radiotherapy status or MGMT methylation status in glioma in CGGA-325 and CGGA-693 cohorts. **(A–D)** Indicates the statistical significance of LOXs mRNA expressions in glioma patients according to stratified analysis by age, gender, radiotherapy status or MGMT methylation status in CGGA-325; **(E–H)** alidation of mRNA expressions of LOXs in glioma patients stratified by age, gender, radiotherapy status or MGMT methylation status in CGGA-693.

Considering the critical role of LOXs in the carcinogenesis and tumor progression (Liburkin-Dan et al., 2022), we firstly explored whether LOXs were associated with the pathological factors of the gliomas, including world health organization (WHO) grade, isocitrate dehydrogenase (IDH) mutation, 1p/19q co-deletion status, age, gender, radiotherapy status and O-6-methylguanine DNA methyltransferase (MGMT) methylation status (Louis et al., 2016). As shown in Figures 2A–E, the elevated expressions of the five members of LOX family were positively correlated with WHO grades in the CGGA-325 cohort. Next, we assessed the relationship of LOXs mRNA expressions and IDH mutation status. It was noteworthy that there were significantly distinct LOXs expressions between wild-type and mutated IDH gliomas in the CGGA-325 dataset. In detail, the glioma patients with IDH mutation exhibited the remarkable decreases of LOXs mRNA expressions (Figure 2F) in this public database. In patients with harboring 1p/19q co-deletion, it was noteworthy that the mRNA expressions of LOXs were significantly diminished (Figure 2G), suggesting reductions of LOXs expressions are highly positively related with 1p/19q co-deletion status. In terms of other stratification factors such as age, gender, radiotherapy status and MGMT methylation status in glioma patients, higher expressions of LOXs were significantly associated with older age and MGMT unmethylated status (Figures 3A,D) while no evident correlation was observed concerning the relationship between LOXs expressions and gender or radiotherapy status (Figures 3B,C). In the meantime, the association of LOXs expressions with pathological features mentioned above was also validated in two other datasets including CGGA-693 and TCGA cohorts. Consistent with the results shown in CGGA-325 dataset, elevations of LOXs expressions were observed in the glioma patients with higher grade, older age or MGMT unmethylated status while the patients with IDH-mutation or 1p/19q co-deletion displayed the reductions of LOXs mRNA expressions (Figures 2H–N and Figures 3E–H in CGGA-693 and Supplementary Figures S2A–E and Supplementary Figures S3A–E in TCGA). Collectively, these results indicate that the expression levels of LOXs have a close relationship with the major clinicopathological characteristics including pathological grades, IDH mutation status, 1p/19q co-deletion status, age, gender and MGMT methylation status.

### Prognostic significance of LOXs in glioma patients

**FIGURE 4:**
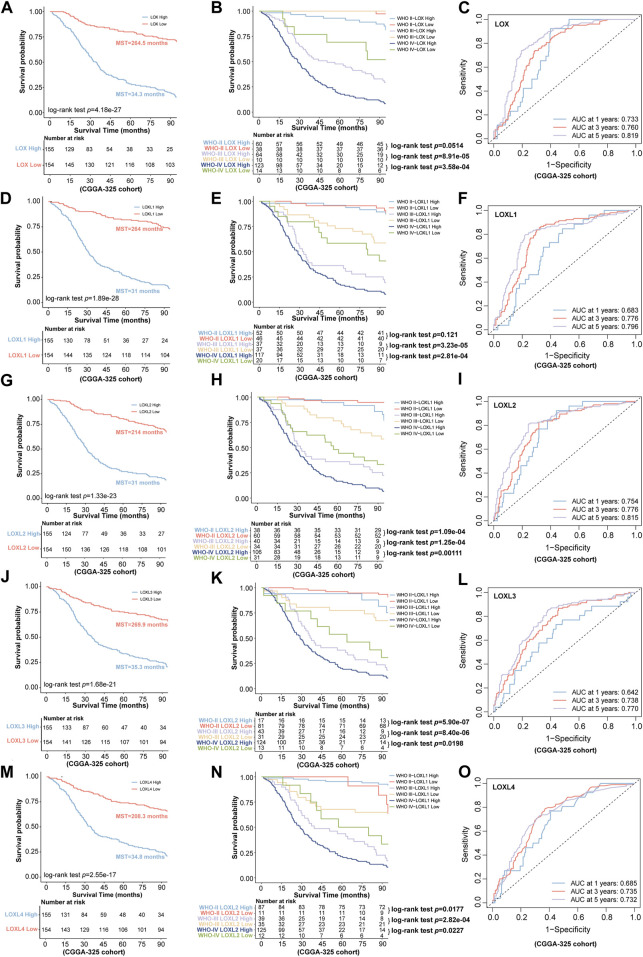
Association of LOXs expressions with prognosis in glioma in CGGA-325 cohort. **(A,D,G,J,M)** the predictive values *via* differential expressions of LOXs including LOX and LOXL1-4; **(B,E,H,K,N)** indicate the patients’ prognosis with differential expressions of LOXs including LOX and LOXL1-4 in different glioma grades; **(C,F,I,L,O)** the predictive value of distinct LOXs expressions in the aspect of 1 year AUC, 3 years AUC and 5 years AUC *via* ROC curve analysis.

**FIGURE 5:**
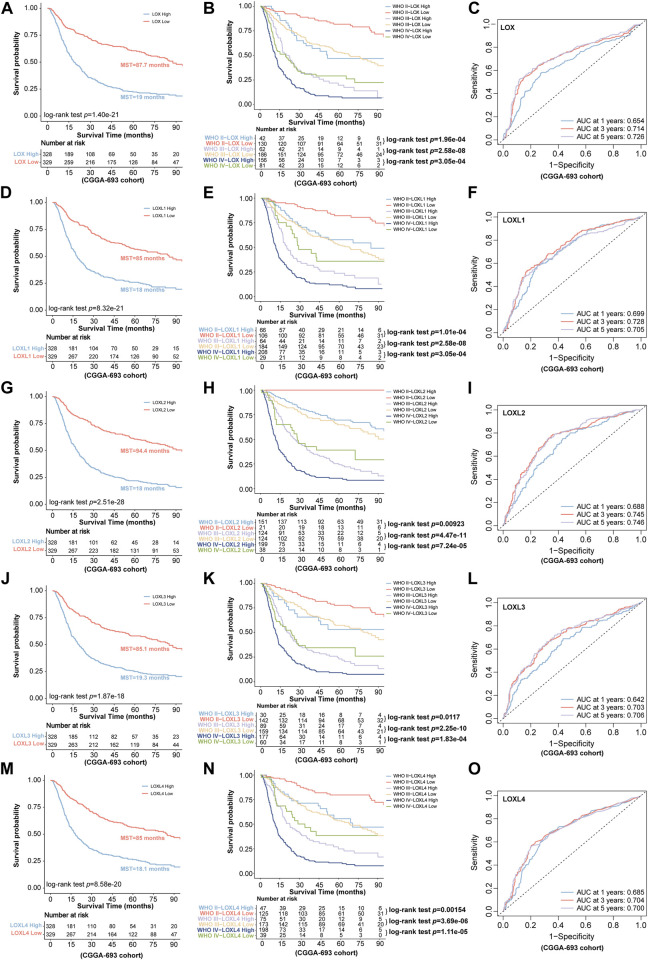
Validation of the predictive value for glioma *via* distinct LOXs expressions in CGGA-693 cohort. **(A,D,G,J,M)** the survival prediction *via* differential expressions of LOXs including LOX and LOXL1-4; **(B,E,H,K,N)** validation of the patients’ prognosis with distinct LOXs expressions in different glioma grades; **(C,F,I,L,O)** evaluation of the predictive value in the aspect of 1 year AUC, 3 years AUC and 5 years AUC *via* ROC curve analysis with distinct LOXs expressions.

Next, we further investigated the prognostic values in various datasets including CGGA-325 and CGGA-693 *via* distinct LOXs expressions. The results shown in CGGA-325 dataset revealed that the glioma patients with the high levels of all members of LOX family including LOX, LOXL1, LOXL2, LOXL3, and LOXL4 displayed poor prognosis with the MST of only 34.3, 31, 31, 35.3, and 34.8 months, respectively (Figures 4A,D,G,J,M). Following the stratified analysis based on WHO grades (including grade II, III, and IV) in CGGA-325 cohort, it was found that the glioma patients with high expression of each member of LOXs displayed shorter overall survival except that no statistical significance was observed following the analysis of distinct LOX or LOXL1 expressions in the WHO grade II glioma patients (Figures 4B,E,H,K,N). ROC curve also indicated that differential LOXs expressions had a good predictive value in the survival time of 1 year, 3 years, or 5 years (Figures 4C,F,I,L,O). In the validation cohort (CGGA-693 dataset), the glioma patients with high levels of LOXs displayed poor prognosis with the MST of only 19, 18, 18, 19.3 and 18.1 months, respectively (Figures 5A,D,G,J,M). In terms of prognosis according to different glioma grades (Figures 5B,E,H,K,N) and ROC curve analysis (Figures 5C,F,I,L,O) of predictive values with distinct LOXs expressions, it was also validated in CGGA-693 dataset although the significance of the prognosis was observed in the grade II glioma patients with high level of LOX or LOXL1 which was not consistent with those findings displayed in CGGA-325 cohort. The results of univariate Cox regression model also revealed that high levels of five members of LOXs all corresponded to large hazard ratios (HRs) in the three datasets (Supplementary Figures S4, S5, S6). Altogether, these results implicate the robust value of distinct LOXs expressions for predicting glioma prognosis.

### LOXs correlated genes and functional enrichment analysis in glioma

**FIGURE 6:**
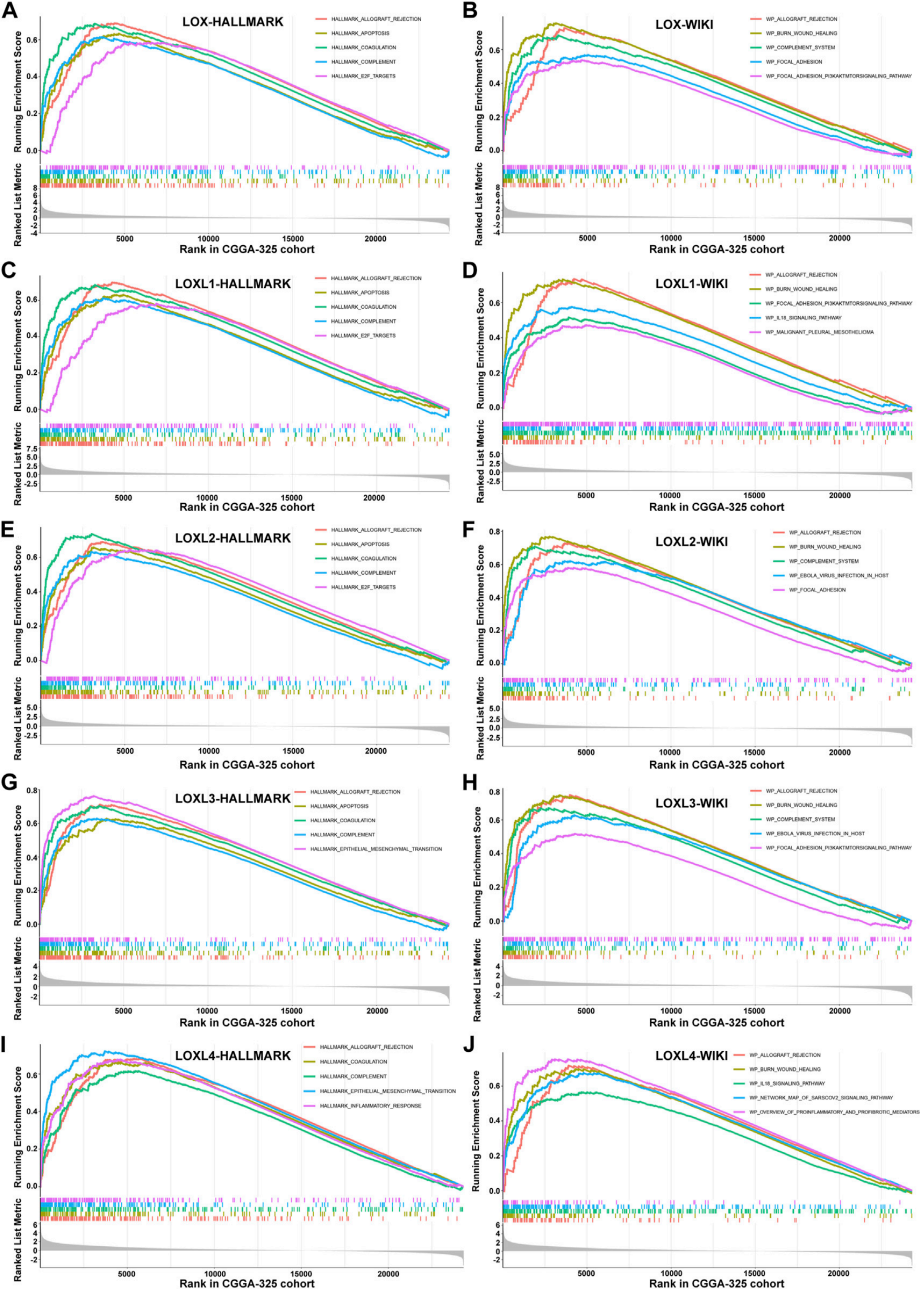
Correlations of LOXs and genes together with functional enrichment analysis in glioma in CGGA-325 cohort. **(A,C,E,G,I)** the top five pathway enrichment with LOXs *via* GSEA-HALLMARK analysis; **(B,D,F,H,J)** indication of the top five pathway enrichment with LOXs *via* GSEA-WIKI analysis.

To clarify the underlying biology associated with differential LOXs expressions, we conducted gene set enrichment analyses (GSEAs) on HALLMARK pathways (Liberzon et al., 2015) and WIKI pathways (Kutmon et al., 2016) for these genes ranked with their gene selection scores (Subramanian et al., 2005) in CGGA-325 cohort. As shown in Figure 6, the top 5 enrichments for each subtype of LOX family enzymes were visualized. The results displayed that LOXs conjointly exhibited the enrichment of the HALLMARK pathways including ALLOGRAFT REJECTION, COAGULATION, and COMPLEMENT. Besides, in the aspect of LOX, LOXL1, and LOXL2, the high expressions of them also had enrichment in APOPTOSIS and E2F-TARGETS while APOPTOSIS and EPITHLIAL MESENCHYMAL TRANSITION were enriched *via* elevated expression of LOXL3. Additionally, the HALLMARK pathways including EPITHLIAL MESENCHYMAL TRANSITION and INFLAMMATORY RESPONSE also belonged to the top 5 enrichment for LOXL4.

The results of WIKI pathway analysis illustrated that LOXs converged in ALLOGRAFT REJECTION and BURN WOUND HEALING as well as inflammation- or immunity-related pathways such as COMPLEMENT SYSTEM (for LOX, LOXL2, and LOXL3), IL18 SIGNALING PATHWAY (for LOXL1 and LOXL4) and OVERVIEW OF PROINFLAMMATORY AND PROFIBROTIC MEDIATORS (for LOXL4) together with the enrichment of viral infection including EBOLA VIRUS INFECTION IN HOST (for LOXL2 and LOXL3) and NETWORK MAP OF SARSCOV2 SIGNALING PATHWAY (for LOXL4). Additionally, the WIKI pathways including FOCAL ADHESION (for LOX and LOXL2), PI3KAKTMTOR SIGNALING PATHWAY (for LOX and LOXL1) and MALIGNANT PLEURAL MESOTHELIOMA (for LOXL1) were also found in the top 5 enrichment. All the HALLMARK and WIKI pathways enriched by LOXs were summarized in Supplementary Table S5.

### Association with LOXs and chemotherapeutic drug sensitivity in glioma

**FIGURE 7:**
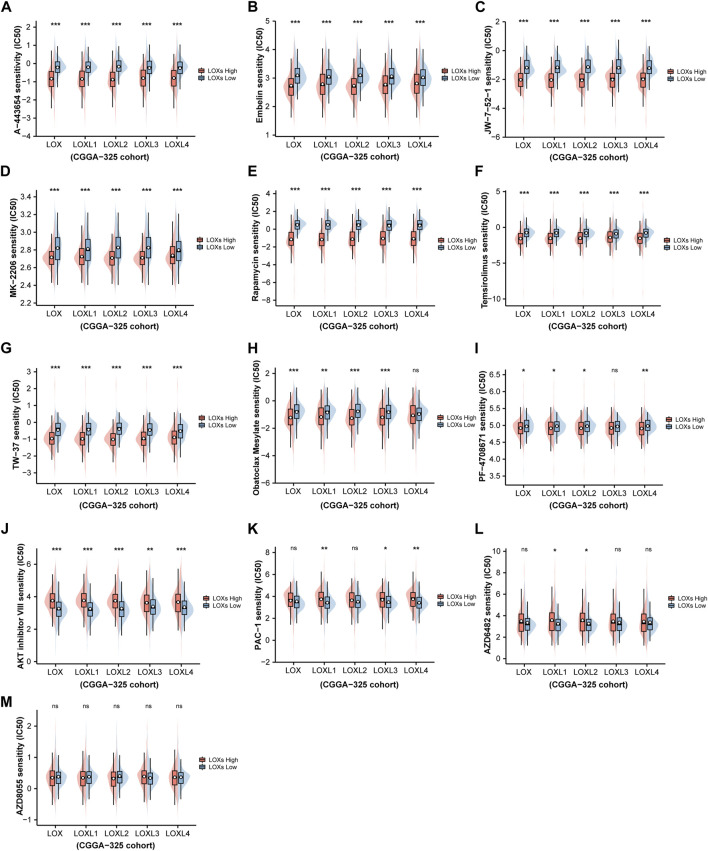
Prediction of chemotherapeutic drug sensitivity in glioma *via* distinct LOXs expressions in CGGA-325 cohort. **(A–I)** Shows the estimated IC50 of nine drugs including A-443654, Embelin, JW-7-52-1, MK-2206, Rapamycin, Temsirolimus, TW-37, Obatoclax mesylate, and PF-4708671 are lower in the high-LOXs expression group compared with low-LOXs expression group; **(J–L)** indicates three drugs including AKT inhibitor VIII, PAC-1, and AZD6482 exhibits higher IC50 in the group with high levels of LOXs expressions; **(M)** no significant difference are observed in the IC50 value of AZD8055 between high-LOXs expression and low-LOXs expression groups.

To further characterize chemotherapeutic drug response in glioma patients *via* differential LOXs expressions, the R package “pRRophetic” (Geeleher et al., 2014) was employed for the estimation of IC50 of the predicted drugs involved in the regulations of the top five pathways (including HALLMARK and WIKI) with distinct LOXs expressions as shown above. It was noteworthy that 13 drugs were predicted between low and high LOXs expressions (Figure 7). The detailed information of these drugs was summarized in Supplementary Table S6. In detail, 12 drugs showed significantly different IC50 *via* distinct expressions of LOXs while no statistical significance was found for AZD8055. Among the 12 drugs affected by differential LOXs expressions, nine drugs including A-443654, Embelin, JW-7-52-1, MK-2206, Rapamycin, Temsirolimus, TW-37, Obatoclax mesylate, and PF-4708671 showed lower IC50 in the high-LOXs expression group compared with low-LOXs expression group while three other drugs including AKT inhibitor VIII, PAC-1 and AZD6482 exhibited higher IC50 in the high levels of LOXs expressions. Collectively, these data suggest that LOXs expressions are good predictive factors of chemotherapeutic drug sensitivity.

### Association with LOXs and immune infiltration in glioma

**FIGURE 8:**
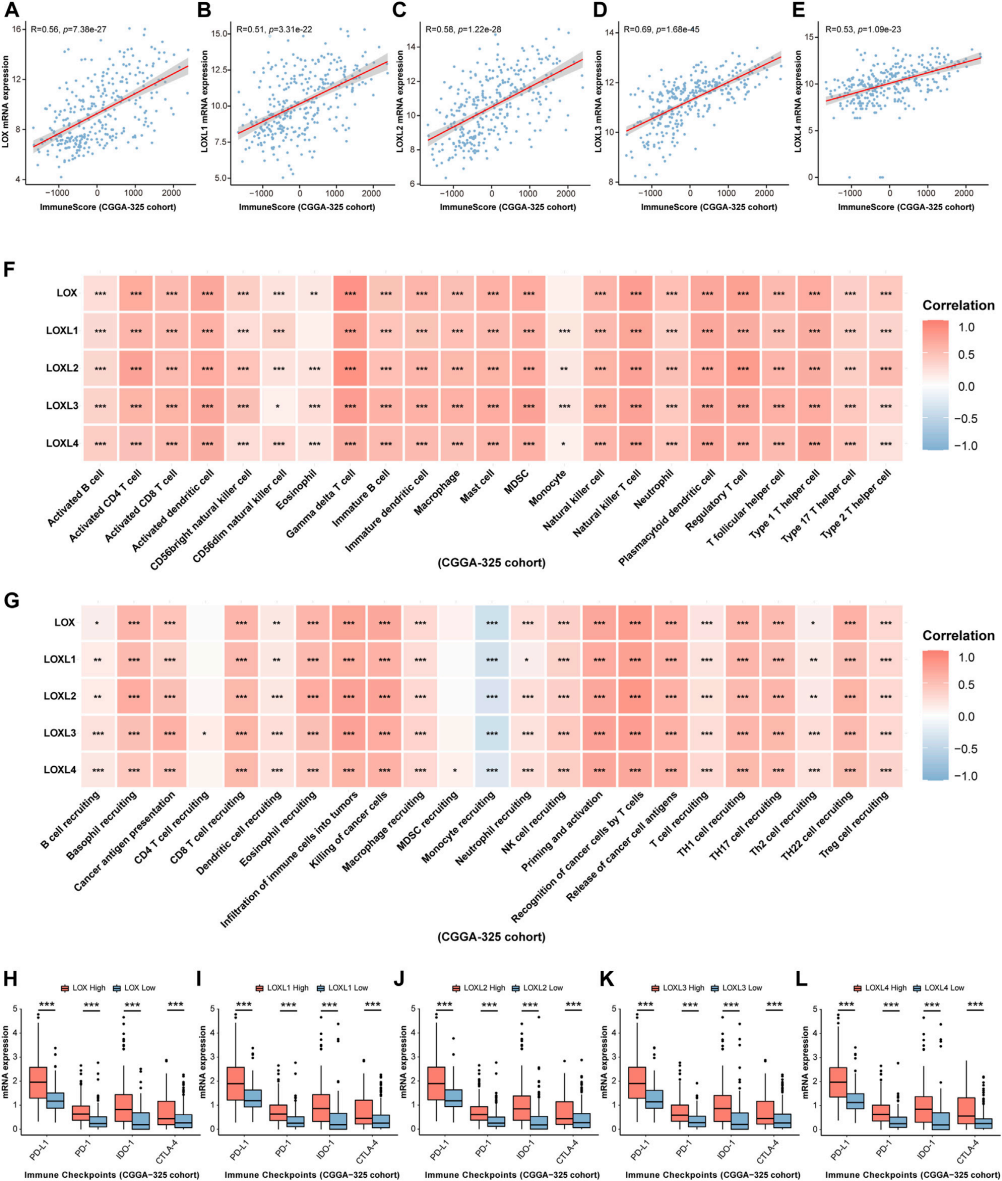
Analysis of the relationship between LOXs expressions and immune response in glioma in CGGA-325 cohort. **(A–E)** Correlation between differential LOXs expressions and immune score in glioma in CGGA-325; **(F,G)** relationship between differential expressions of LOXs and immune infiltration score or cancer immunity cycles in glioma in CGGA-325; **(H–L)** relationship between distinct LOXs expressions and immune checkpoints (including PD-L1, PD-1, IDO-1, and CTLA-4) in glioma in CGGA-325.

**FIGURE 9:**
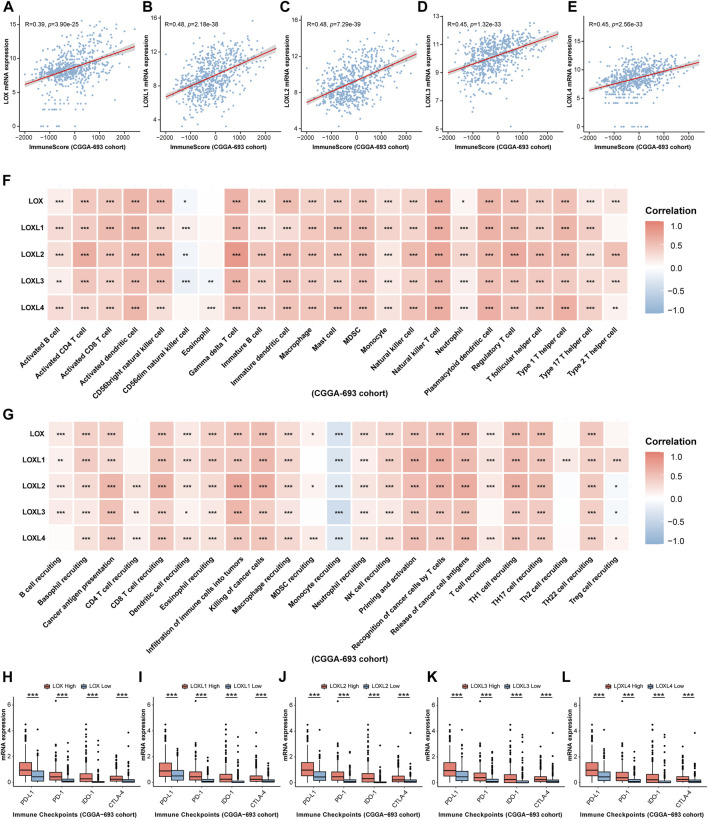
Validation of the relationship between LOXs expressions and immune response in glioma in CGGA-693 cohort. **(A–E)** Correlation between differential LOXs expressions and immune score in glioma in CGGA-693; **(F,G)** relationship between differential expressions of LOXs and immune infiltration score or cancer immunity cycles in glioma in CGGA-693; **(H–L)** relationship between distinct LOXs expressions and immune checkpoints (including PD-L1, PD-1, IDO-1, and CTLA-4) in glioma in CGGA-693.

Considering some previous investigations illustrating that LOXs, especially, LOX, LOXL1, and LOXL4 are closely associated with immunity (Tenti and Vannucci, 2020; Li et al., 2021; Tan et al., 2021), finally contributing to tumor progression, we would like to explore whether LOXs expressions were correlated with glioma immunity. As displayed in Figures 8A–E, ESTIMATE analysis demonstrated that immune score was positively correlated with high expression of each subtype of LOXs in CGGA-325 cohort. These results were also validated in CGGA-693 (Figures 9A–E) and TCGA (Supplementary Figures S7A–E) datasets. Analysis of immune cell infiltration revealed that innate immune cell infiltration including natural killer cell, macrophage, mast cell, MDSC, plasmacytoid dendritic cell was abundant in CGGA-325 (Figure 8F), CGGA-693 (Figure 9F) and TCGA (Supplementary Figure S7F) cohorts. The cancer immunity cycle is critical for assessing anticancer immune response (Chen and Mellman, 2013). It was observed that in the LOXs-high expression group, activations of various steps of immunity cycles were found, which included antigen release by cancer cells (step 1); cancer antigen processing and presentation (step 2); T cell initiation and activation (step 3); T cells trafficking to cancer lesions (step 4); T cells infiltration into tumors (step 5); recognition of cancer cells by T cell (step 6) and killing of cancer cells (step 7) in CGGA-325 (Figure 8G), CGGA-693 (Figure 9G) and TCGA (Supplementary Figure S7G) cohorts. Besides, we also made an analysis of the relationship between differential LOXs expressions and the expressions of immune checkpoints including PD-L1, PD-1, IDO-1, and CTLA4 in glioma. In CGGA-325 cohort, it was intriguing that these immune checkpoints were all abundantly elevated with high LOXs expressions (Figures 8H–L). Consistently, in CGGA-693 (Figures 9H–L) and TCGA (Supplementary Figures S8A–E) cohorts, it was also noted that the patients with high LOXs expressions exhibited the high levels of PD-L1, PD-1, IDO-1, and CTLA4. Altogether, these results again verify the significance of the prediction of glioma immunotherapy *via* differential LOXs expressions.

### Validation of the significant roles of LOXs in tumor immunotherapy

**FIGURE 10:**
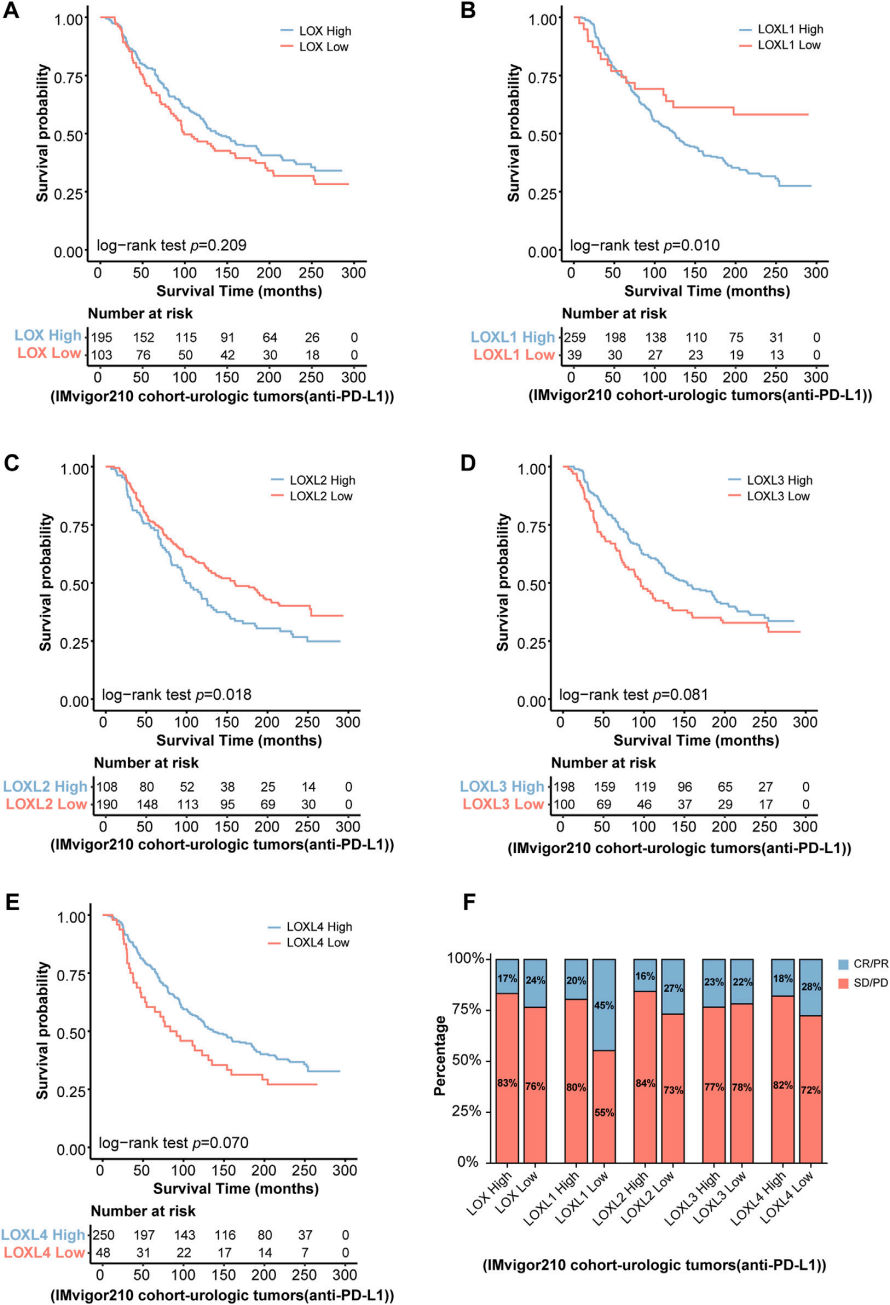
Validation of the predictive roles of LOXs in tumor immunotherapy in IMvigor 210 cohort. **(A–E)** Indicates the patients’ survival following anti-PD-L1 treatment according to distinct LOXs expressions in IMvigor 210 cohort; **(F)** shows the fraction of urologic tumor patients with response to anti-PD-L1 therapy in high- and low-LOXs subgroups of IMvigor 210 cohort. CR, complete response; PR, partial response; SD, stable disease; PD, progressive disease.

Due to the critical roles of LOXs in remodeling immune microenvironment, we wonder whether the predictive values of LOXs in tumor immunotherapy were validated in clinical practice. Since the clinical cohort of glioma immunotherapy was scarce, the two well-known clinical cohorts including IMvigor 210 (urologic tumor patients treated with anti-PD-L1therapy) (Zhao et al., 2021) and Van Allen 2015 (melanoma patients treated with anti-CTLA-4 therapy) (Tu et al., 2022), which were previously used for assessing the relationship between target gene expressions and glioma immunotherapy, were selected in our present work. KM survival analysis revealed that the patients with differential LOXs expressions were associated with distinct clinical outcomes in IMvigor210 cohort, with the high-LOXL1 or high-LOXL2 subgroup showing poor response to anti-PD-L1 therapy (Figures 10B,C,F), despite no significance was found in terms of differential LOX (Figure 10A), LOXL3 (Figure 10D) or LOXL4 (Figure 10E) expressions. However, in another immunotherapy cohort Van Allen 2015 (anti-CTLA4 therapy), the patients with the high-LOXL3 (Supplementary Figures S9D, S10D) or high-LOXL4 (Supplementary Figures S9E, S10E) subgroup exhibited poor response to anti-CTLA4 therapy although no significance was observed in differential LOX (Supplementary Figures S9A, S10A), LOXL1 (Supplementary Figures S9B, S10B) or LOXL2 (Supplementary Figures S9C, S10C) expression groups. Taken together, these results indicate the strong correlation with differential LOXs expressions and responsiveness to immunotherapy, which may serve as critical factors for predicting clinical prognosis of cancer patients.

### Experimental verification of mRNA and protein expressions of LOXs in glioma cell lines

**FIGURE 11:**
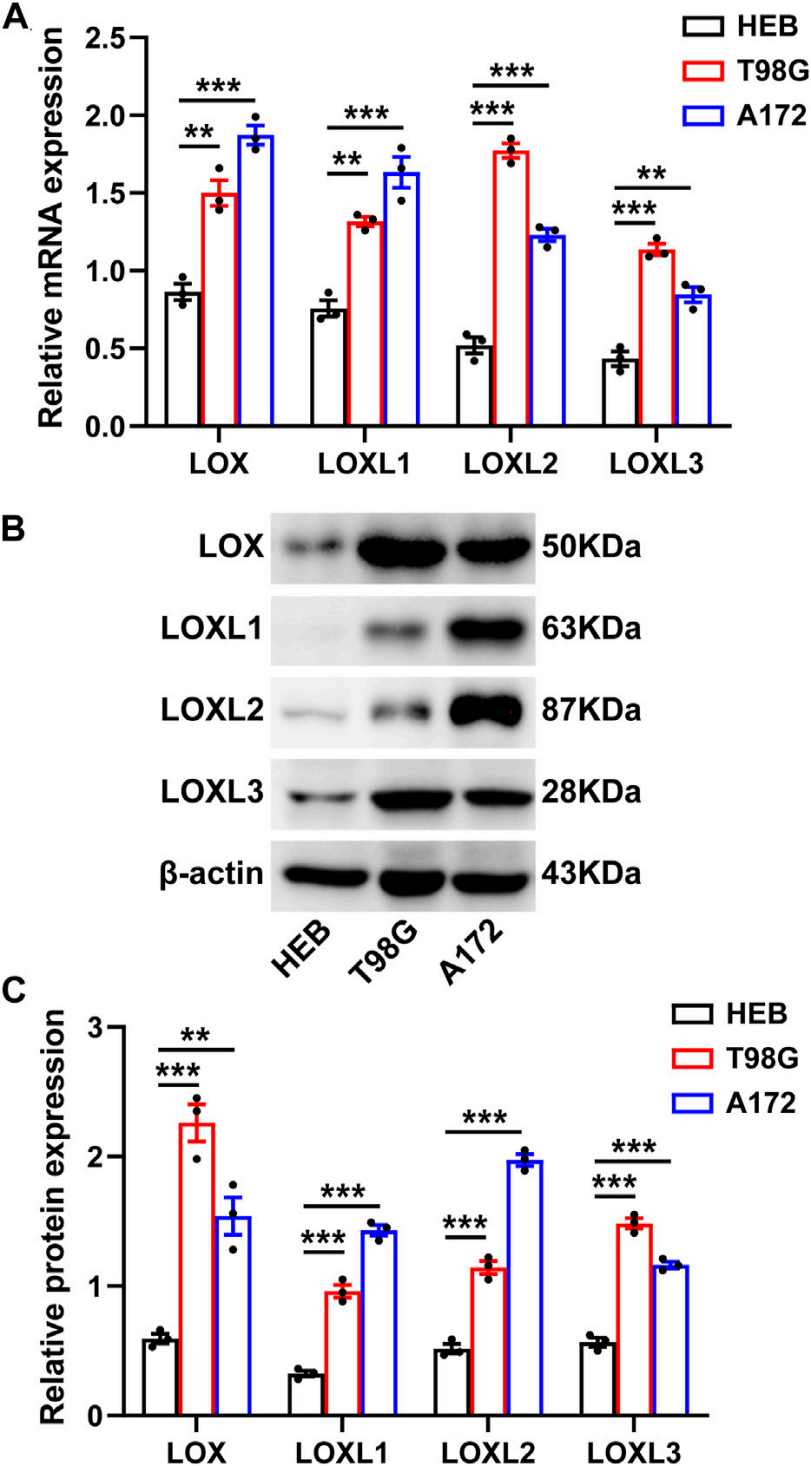
Experimental validation of the differential LOXs expressions at mRNA and protein levels in glioma cells. **(A)** shows mRNA expressions of LOXs including LOX and LOXL1-3 in glioma cells; **(B)** representative protein bands of LOXs in glioma cells; **(C)** statistical analysis of protein expressions of LOXs in glioma cells.

Finally, RT-qPCR and Western blot analysis were carried out to validate the mRNA and protein levels of LOXs including LOX, LOXL1, LOXL2, LOXL3, and LOXL4 in glioma cells (T98G and A172) and the normal cell line (HEB). The results indicated that the mRNA (Figure 11A) and protein expressions (Figures 11B,C) of LOX, LOXL1, LOXL2, and LOXL3 were significantly upregulated in these glioma cells than in HEB cell line while the expressions of LOXL4 at mRNA and protein level were not determined in these cell lines (data not shown). Collectively, these results, especially the mRNA expressions of LOX, LOXL1, LOXL2, and LOXL3 support those obtained *via* our bioinformatics analysis (Figures 11A–C).

## Discussion

The present work focused on the comprehensive analysis of LOXs expressions in the predictions of prognosis, chemotherapy and immunotherapy in glioma. The major findings of our present study included at least four items shown as follows: 1) High gene expressions of LOXs were positively correlated with glioma grades while elevations of this family have a negative association with IDH mutation or 1p/19q co-deletion; 2) The glioma patients with high LOXs expressions exhibited poorer prognosis than those with low LOXs expressions; 3) Differential levels of LOXs expressions had significant potential for the prediction of chemotherapeutic drug sensitivity; 4) Immune signaling was activated in the high LOXs expressions group, which suggests that the glioma patients with high LOXs expressions may obtain the satisfactory effects *via* immunotherapy; 5) Experimental results showed that various LOXs especially LOX, LOXL1, LOXL2, and LOXL3 were highly expressed in glioma cell lines at mRNA and protein levels.

Extracellular proteins hold great promise in curing human diseases including cancers. LOXs, well-known secreted amine oxidases, have been extensively discussed in modulating tumor initiation and progression. In glioma research, there are a plethora of studies supporting this notion. For instance, a previous study has demonstrated that the highest LOXL3 expression level is observed in the glioblastoma and genetic silencing of LOXL3 in U87 glioma cells has pronounced inhibition on invasive phenotype (Laurentino et al., 2021), which is a pivotal factor contributing to the recurrence and poor prognosis of GBM patients. Meanwhile, LOXL1 is also previously demonstrated to exhibit antiapoptotic property and promote gliomagenesis (Yu et al., 2020). Li et al. (2018) has revealed that LOXL1 is highly expressed in tissue samples of malignant gliomas and LOXL1 accelerates cell proliferation *via* Wnt/β-catenin signaling. In respect of LOX, one of the major subtypes of LOXs, it has been demonstrated that it is positively correlated with the malignant grade of astrocytomas (da Silva et al., 2015). The experimental results in our present work demonstrated the elevations of LOX, LOXL1, LOXL2, and LOXL3 in various glioma cell lines. It suggests that these isoforms of LOXs may serve as contributing factors for triggering glioma progression. Furthermore, GBM patients harboring IDH1 mutation display lower expression of LOX in the nucleus. Results from our present work revealed that LOXs were increased with the degree of malignancy and the glioma patients with IDH mutation exhibited the decrease of LOXs expression, which are consistent with the previous investigations. In addition, our present study also disclosed that diminished expression levels of LOXs were observed in the gliomas with 1p/19q codeletion. These data altogether imply that LOXs are critical for regulating clinicopathological features in glioma at least including WHO grades, IDH mutation status and 1p/19q codeletion status.

It is well known that carcinogenesis and tumor progression are always associated with patients’ prognosis (Weller et al., 2015). The more dismal prognosis always corresponds to greater grade of malignancy. Our current findings showing the glioma patients with the higher levels of LOXs expressions exhibited the shorter MST. It indicates the poorer clinical outcome in glioma patients with high LOXs expressions. With respect of IDH mutation status or 1p/19q codeletion status, it has been reported that the glioma patients with IDH mutation or 1p/19q codeletion always exhibit more satisfactory prognosis (Alentorn et al., 2014; Brat et al., 2015). Indeed, the results of our present work illustrated that the glioma patients harboring IDH mutation or 1p/19q codeletion displayed lower LOXs expressions and better prognosis than those with wild type or 1p/19q non-codeletion. These results again support the notion that differential LOXs expressions are critical factors affecting glioma progression and patients’ clinical outcome. The contribution of LOXs to glioma cell biology may involve several pathway mechanisms. For example, it has been demonstrated that LOX functions as a macrophage chemoattractant to facilitate glioma progression *via* activating PYK2 signaling (Chen et al., 2019). In addition, LOXL1 is also found to inhibit apoptosis in glioma *via* stabilizing BAG family molecular chaperone regulator 2 (Yu et al., 2020).

Chemotherapy is the first-line regimen for the treatment of various carcinomas including glioma. Nowadays, temozolomide has been successfully approved for treating glioma patients (Friedman et al., 2000). However, temozolomide resistance is very prevalent following chemotherapy in glioma. It is estimated that the efficacy of TMZ has been present in only 46% of glioma patients (García et al., 2009). Given that the critical roles of LOXs in glioma, exploration of the predictive value of LOXs in chemotherapeutic drug sensitivity has great therapeutic potential. In our current work, there are 12 drugs predicted by differential LOXs expressions, among which nine drugs (A-443654, Embelin, JW-7-52-1, MK-2206, Rapamycin, Temsirolimus, TW-37, Obatoclax mesylate, and PF-4708671) showed lower IC50 in the high-LOXs expression groups. In contrast, three other drugs (AKT inhibitor VIII, PAC-1, and AZD6482) exhibited higher IC50 in the high-LOXs expression groups. These findings indicate that distinct LOXs expressions have predictive significance for chemotherapeutic drug sensitivity.

Immunotherapy has been effective against a variety of cancers. Nowadays, there are several well-established immunotherapeutic approaches to activate antitumor immunity, which includes monoclonal antibodies, immune adjuvants and vaccines (Dougan and Dranoff, 2009). In glioma research, current immune-associated treatment regimens including vaccination, oncolytic viruses, immune checkpoint inhibitors, and chimeric antigen receptor T cell therapy are applied for clinical trials (Lim et al., 2018). These data altogether indicate the critical role of immunity in glioma progression. In our present work, distinct LOXs expressions had a close relationship with immune signaling. In detail, it was found that the fraction of immune cell infiltration and the expression levels of diverse immune checkpoints like PD-L1, PD-1, CTLA-4, and IDO-1 were elevated in high-LOXs expressions group. It suggests the association of LOXs expression and glioma immunity. In fact, there is unequivocal evidence supporting manipulation of LOXs have significant effects on cancer immunity. Recent investigations have depicted that high level of LOX in the CD^8+^ T cells promotes paclitaxel (PTX) chemotherapy-induced metastatic process (Haj-Shomaly et al., 2022). LOX inhibition counteracts the metastasis-promoting effect of PTX. In addition, epigenetic activation of LOX *via* lncRNA/miR-29c has also been demonstrated to facilitate M2 macrophage polarization and tumor immune escape, finally rendering tumor cells resistant to chemotherapeutic drugs in gastric cancer (Nai et al., 2021). In addition, LOXL3 (Wang et al., 2021), and LOXL4 (Tan et al., 2021) are also reported to facilitate immune evasion and then result in hepatocarcinogenesis. Results from our present work revealed that the predictive roles of LOXs expression in tumor immunotherapy were also confirmed in two immunotherapy cohorts including IMvigor 210 (anti-PD-L1 therapy) and Van Allen 2015 (anti-CTLA4 therapy). Taken together, these findings support that differential LOXs have the great potential for predicting glioma immunotherapy.

Of course, there are also other items required to be clarified in the future investigations. First, the major findings of our present are based on the public database and some simple experiments. The biological function of LOXs in glioma is essential. Second, the molecular mechanism by which each type of LOXs regulates glioma initiation, glioma progression, chemotherapeutic drug sensitivity and immunotherapy required to be explored in the coming years. In any case, our present work provides the comprehensive analysis of the roles of LOXs in the predictions of prognosis, chemotherapy and immunotherapy in gliomas. From the translational aspect in clinical settings, LOXs may serve as the promising biomarkers for the personalized glioma therapy.

## Data Availability

The datasets presented in this study can be found in online repositories. The names of the repository/repositories and accession number(s) can be found in the article/Supplementary Material.

## Supplementary material

SUPPLEMENTARY TABLE S1 Clinical characteristics of glioma patients in CGGA-325, CGGA-693 and TCGA cohorts.

SUPPLEMENTARY TABLE S2 Basic information of datasets verifying the relationship between LOXs and immunotherapy.

SUPPLEMENTARY TABLE S3 Survival information for datasets selected in this study.

SUPPLEMENTARY TABLE S4 Primer sequences used in our present study.

SUPPLEMENTARY TABLE S5 Detailed information of LOXs-enriched HALLMARK and WIKI pathways.

SUPPLEMENTARY TABLE S6 Prediction of chemotherapeutic drug according to LOXs associated top 5 HALLMARK and WIKI pathways.

SUPPLEMENTARY FIGURE S1 Summarization of the purpose of data analysis and the corresponding package.

SUPPLEMENTARY FIGURE S2 Relationship between LOXs expressions and WHO grades in glioma in TCGA cohort.

SUPPLEMENTARY FIGURE S3 Relationship between LOXs expressions and IDH mutation status, 1p/19q co-deletion status, age, gender or radiotherapy status in glioma in TCGA cohort.

SUPPLEMENTARY FIGURE S4 Forest plot showing the effects of differential LOXs expressions for each subtype of LOXs on patients’ prognosis in CGGA-325 cohort.

SUPPLEMENTARY FIGURE S5 Forest plot showing the effects of differential LOXs expressions for each subtype of LOXs on patients’ prognosis in CGGA-693 cohort.

SUPPLEMENTARY FIGURE S6 Forest plot showing the effects of differential LOXs expressions for each subtype of LOXs on patients’ prognosis in TCGA cohort.

SUPPLEMENTARY FIGURE S7 Relationship between LOXs expressions and immune response in glioma in TCGA cohort.

SUPPLEMENTARY FIGURE S8 Relationship between LOXs expressions and immune checkpoints in glioma in TCGA cohort.

SUPPLEMENTARY FIGURE S9 Prognosis evaluation of patients following immunotherapy (anti-CTLA4 therapy) *via* differential LOXs expressions in Van Allen 2015 cohort.

SUPPLEMENTARY FIGURE S10 Analysis of the fraction of melanoma patients with response to immunotherapy (anti-CTLA4 therapy) in high- and low-LOXs subgroups of Van Allen 2015 cohort.

## Glossary

LOXs: lysyl oxidase family enzymes
CGGA: Chinese Glioma Genome Atlas
TCGA: the Cancer Genome Atlas
LGG: lower-grade glioma
HGG: high-grade glioma
GBM: glioblastoma
MST: median survival time
IDH: isocitrate dehydrogenase
1p/19q: the short arm of chromosome 1 and the long arm of chromosome 19
TME: tumor microenvironment
ECM: extracellular matrix
LOXL: LOX-like
FPKM: fragments per kilobase of transcript per million reads sequenced
TPM: transcripts per kilobase million
WHO: World Health Organization
KM: Kaplan-Meier
ROC: receiver operating characteristic
AUC: areas under curve
GSEA: gene set enrichment analysis
ES: enrichment score
NES: normalization of enrichment scores
FDR: false discovery rate
GDSC: Genomics of Drug Sensitivity in Cancer
IC50: half-maximal inhibitory concentration;
TIME: tumor immune microenvironment
ssGSEA: single sample gene set enrichment analysis
GSVA: gene set variation analysis
TIP: Tumor Immunophenotype
FBS: fetal bovine serum
RT-qPCR: real-time quantitative polymerase chain reaction
RNA: ribonucleic acid
cDNA: complementary DNA
DNA: deoxyribonucleic acid
TBST: Tris-buffered saline with Tween-20
OS: overall survival
HRs: hazard ratios
mRNA: messenger RNA
MGMT: O-6-methylguanine DNA methyltransferase
PD-L1: programmed cell death-ligand 1
PD-1: programmed death 1
IDO-1: indoleamine 2,3-dioxygenase 1
CTLA-4: cytotoxic T-lymphocyte-associated protein 4
PTX: paclitaxel
