# Supplementary figures and images for: Identification and validation of roles of lysyl oxidases in the predictions of prognosis, chemotherapy and immunotherapy in glioma
Source: Front Pharmacol. 2022 Aug 31;13:990461. doi: 10.3389/fphar.2022.990461 (PMC9490755; doi:10.3389/fphar.2022.990461)

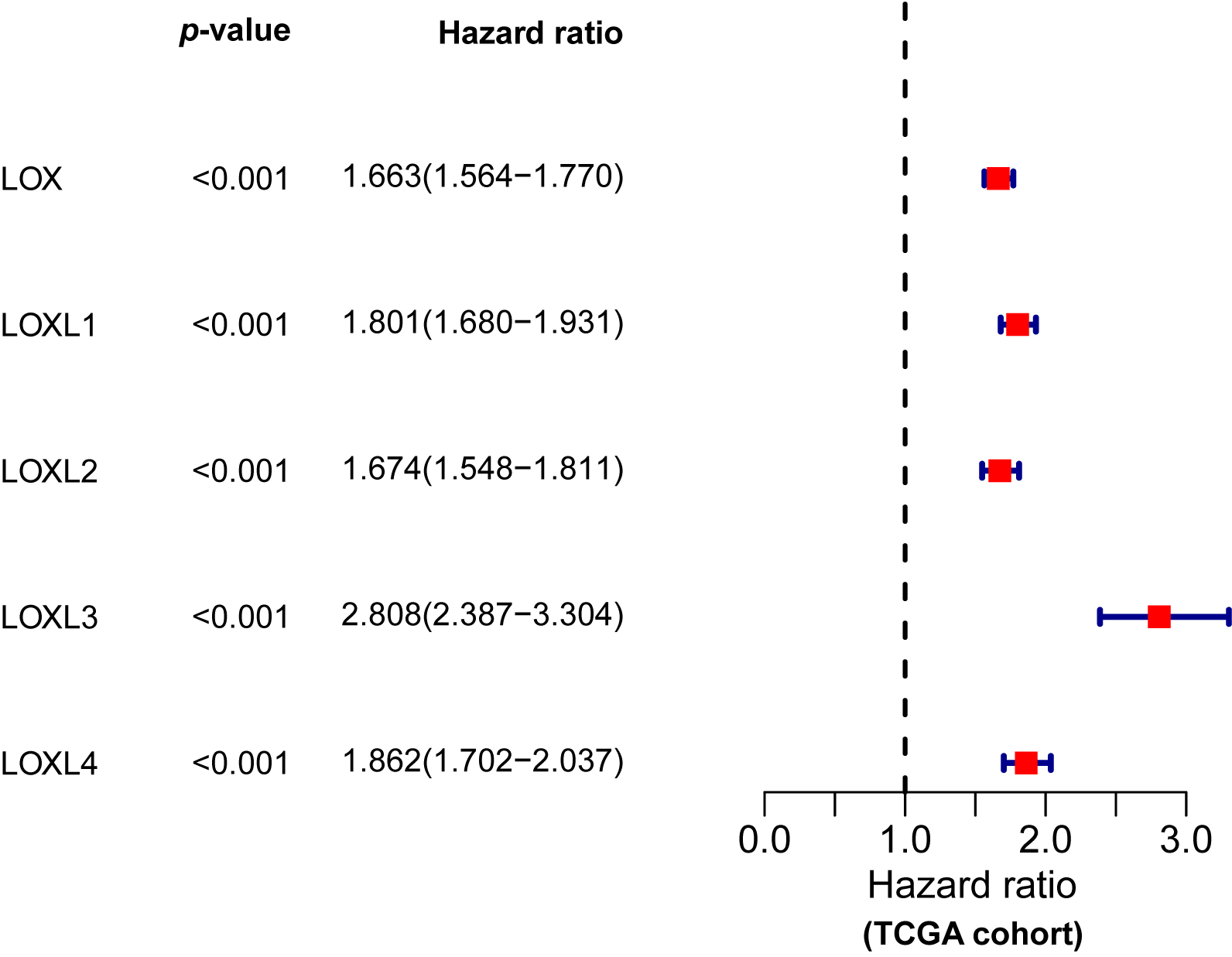

Supplement: Supplementary file 1 [file Image6.TIF]

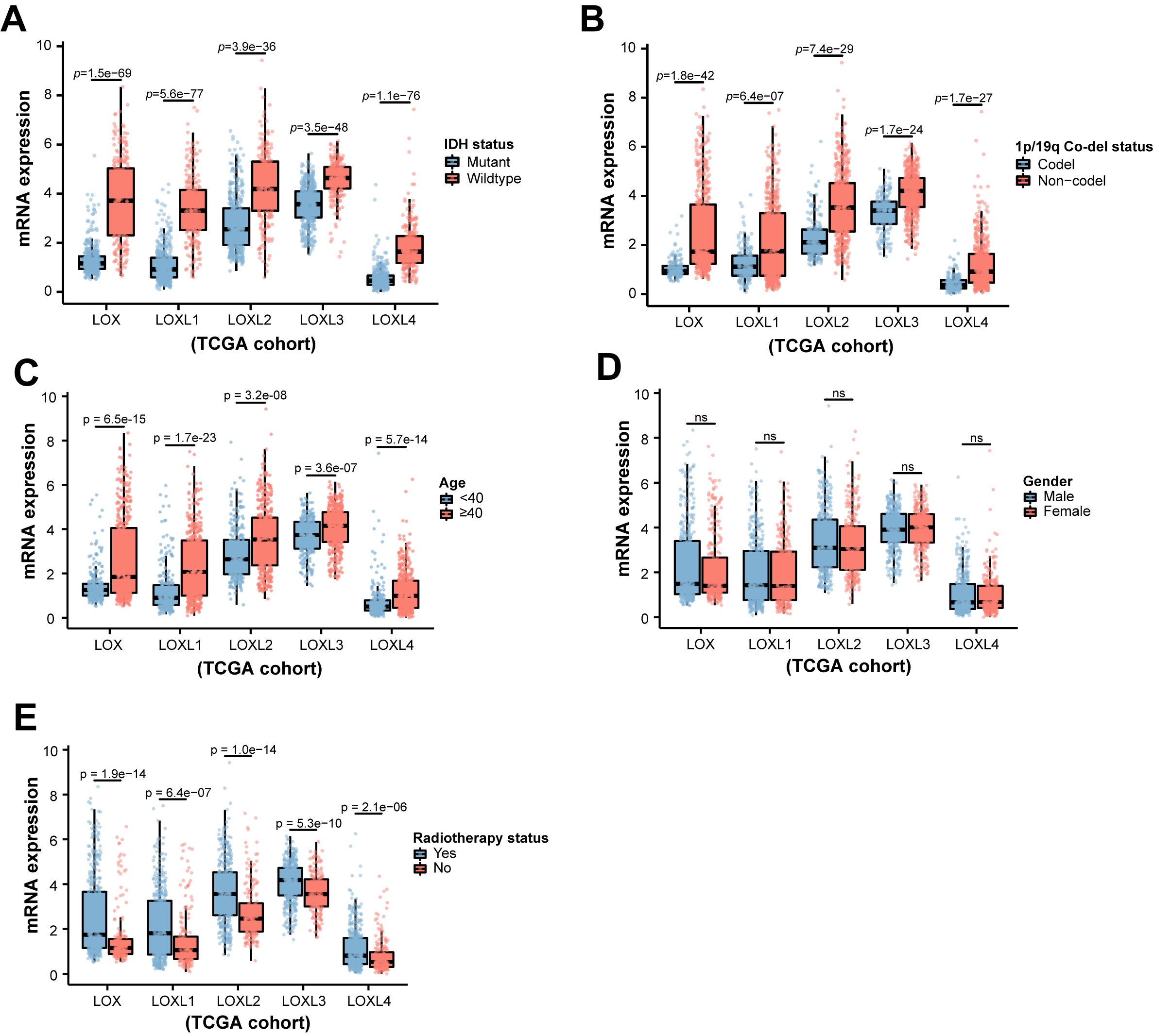

Supplement: Supplementary file 2 [file Image3.TIF]

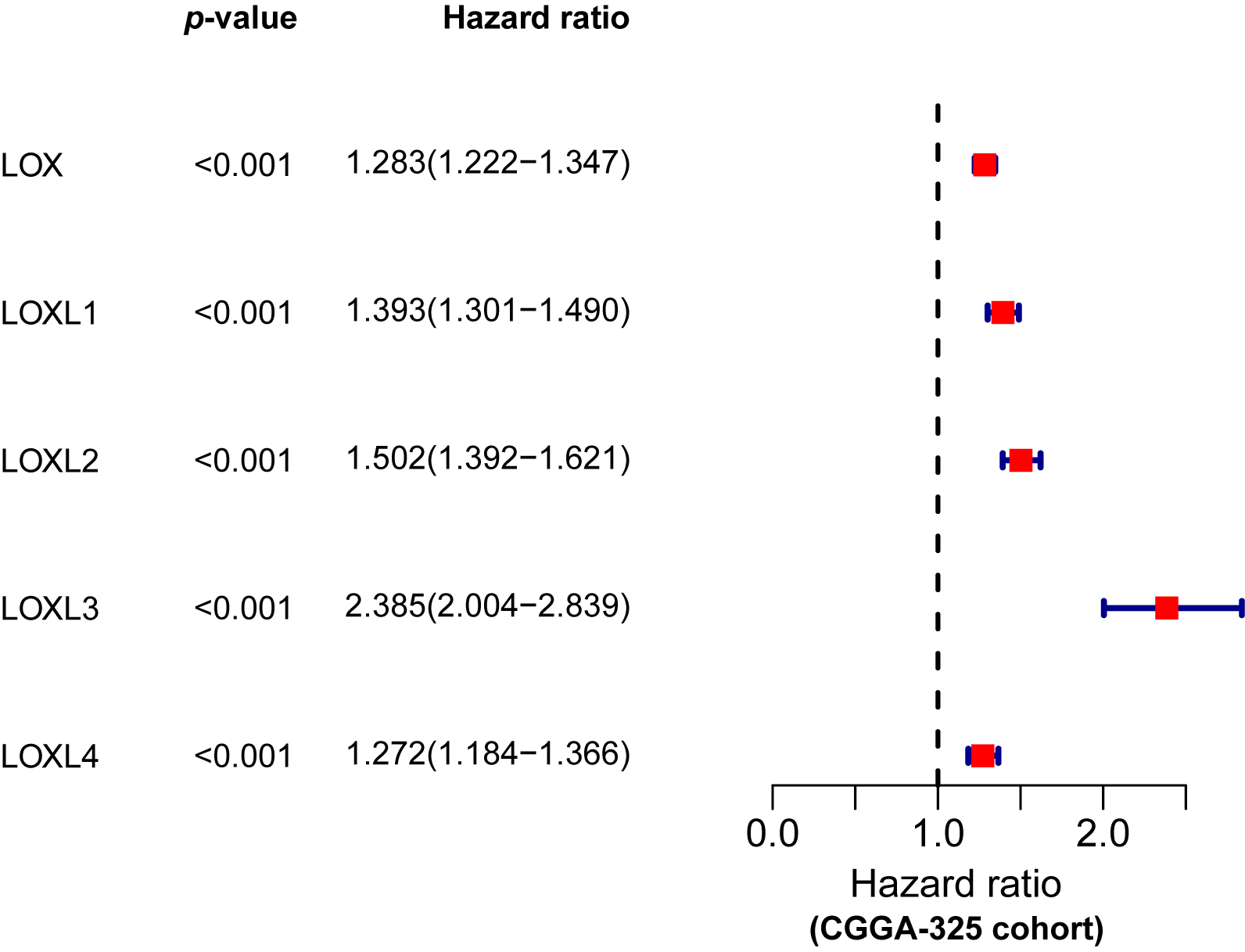

Supplement: Supplementary file 3 [file Image4.TIF]

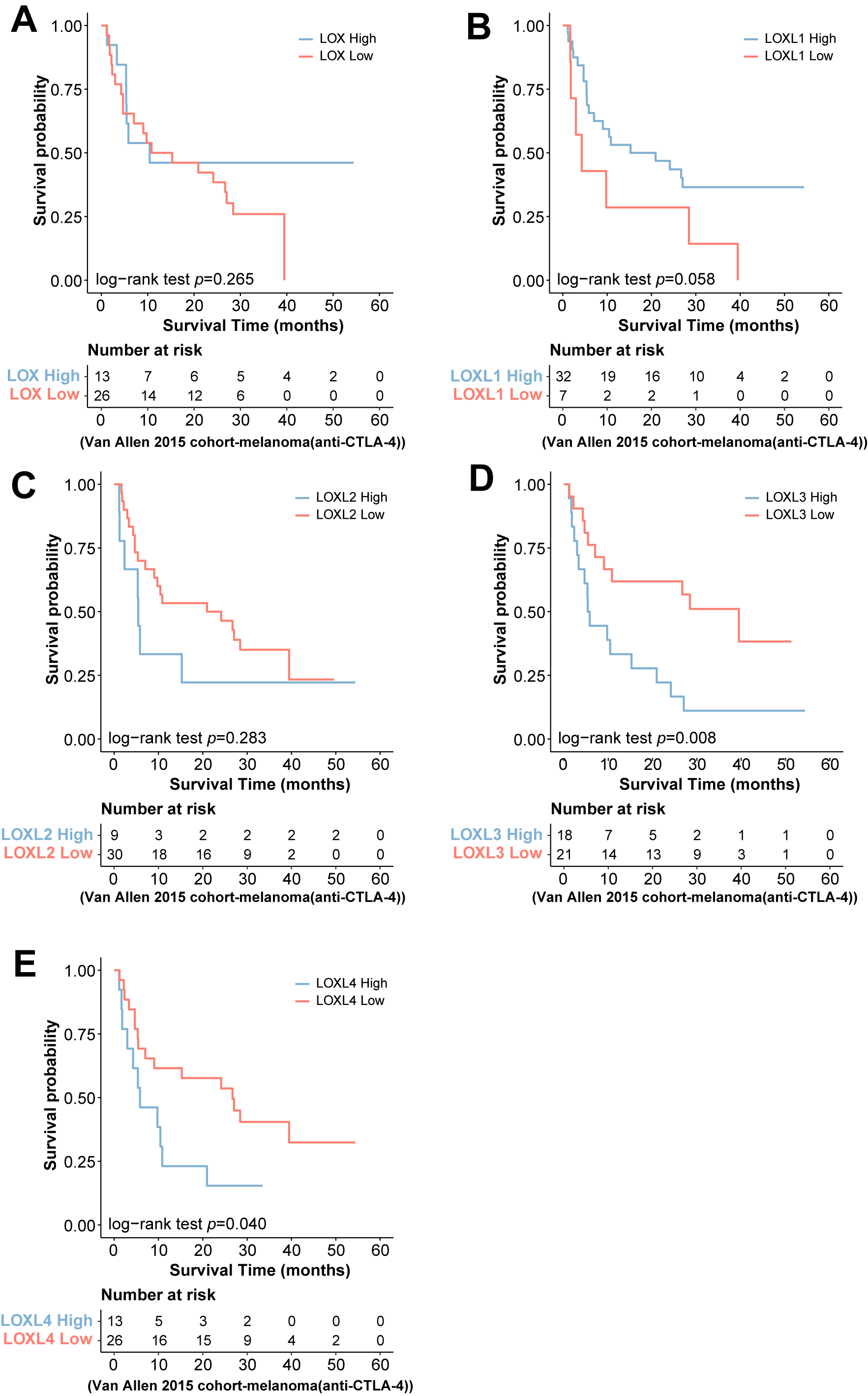

Supplement: Supplementary file 4 [file Image9.TIF]

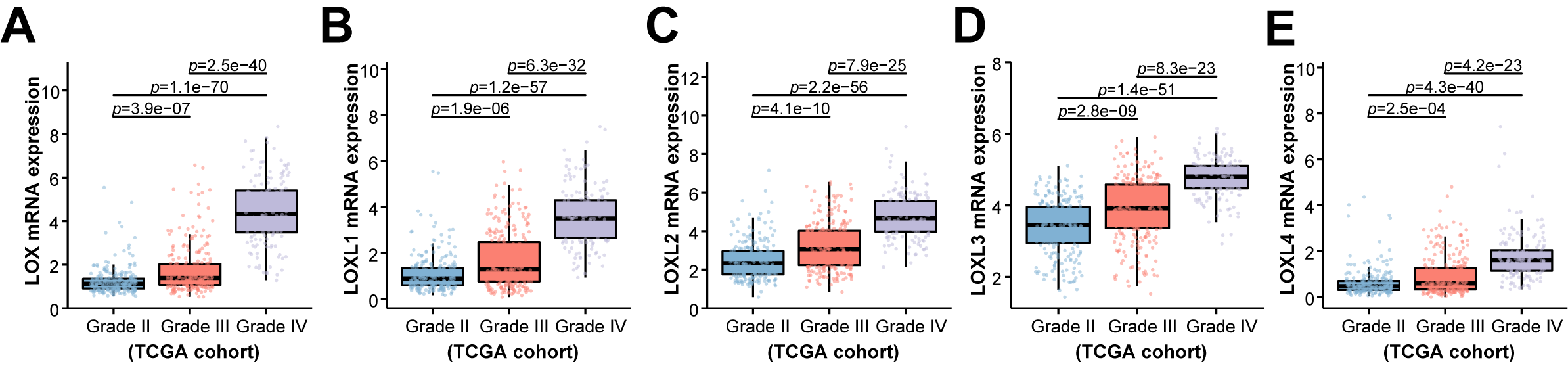

Supplement: Supplementary file 5 [file Image2.TIF]

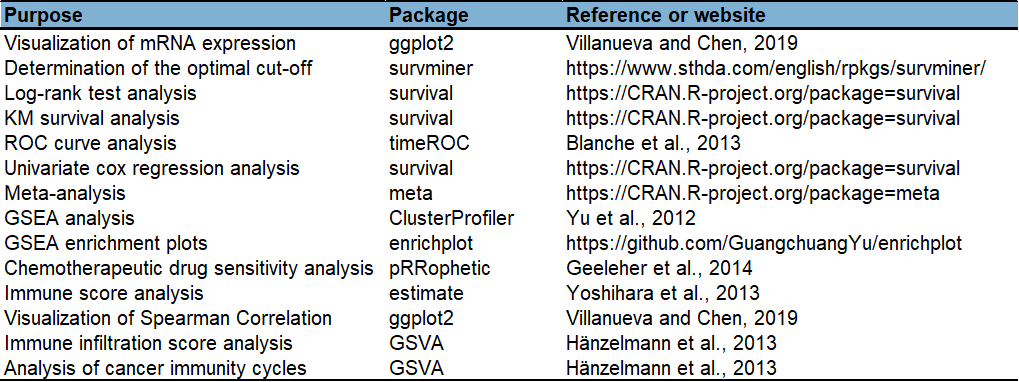

Supplement: Supplementary file 6 [file Image1.TIF]

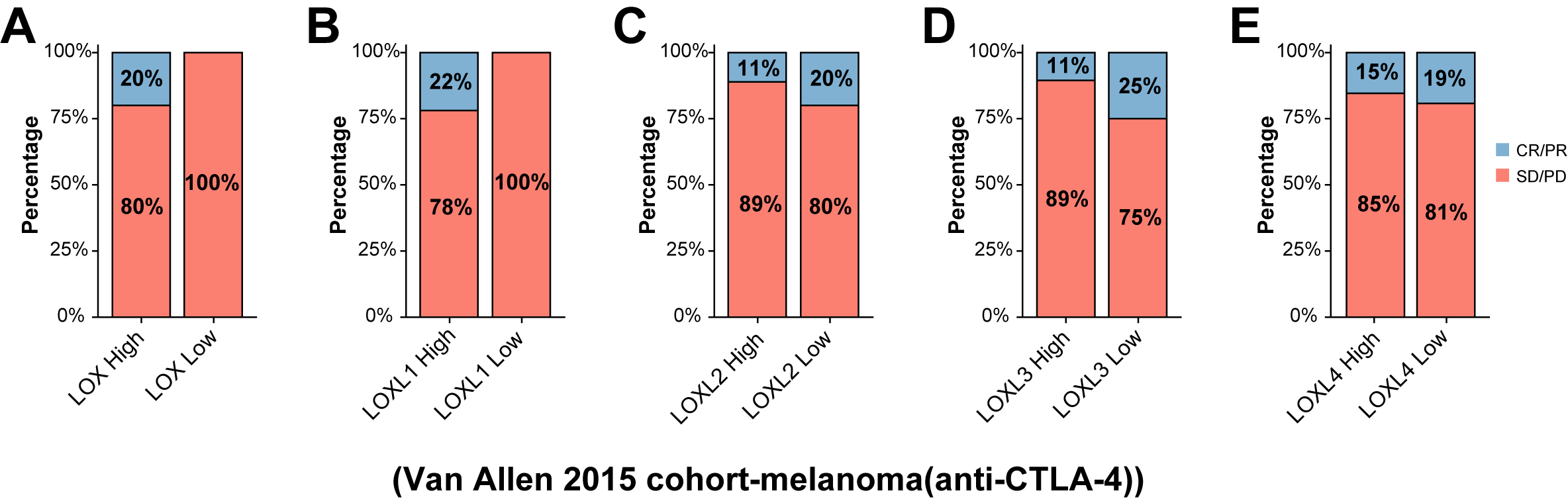

Supplement: Supplementary file 7 [file Image10.TIF]

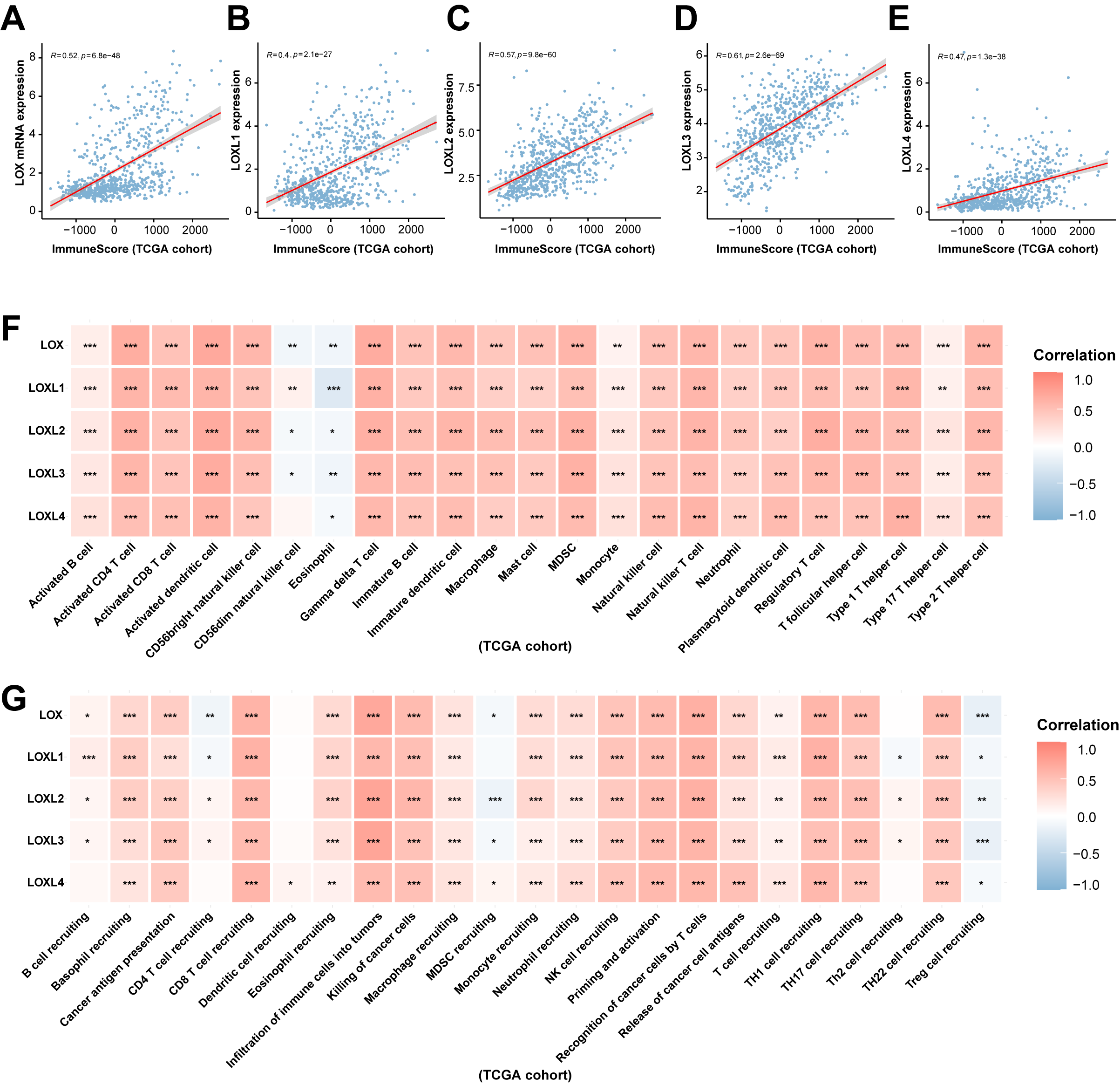

Supplement: Supplementary file 8 [file Image7.TIF]

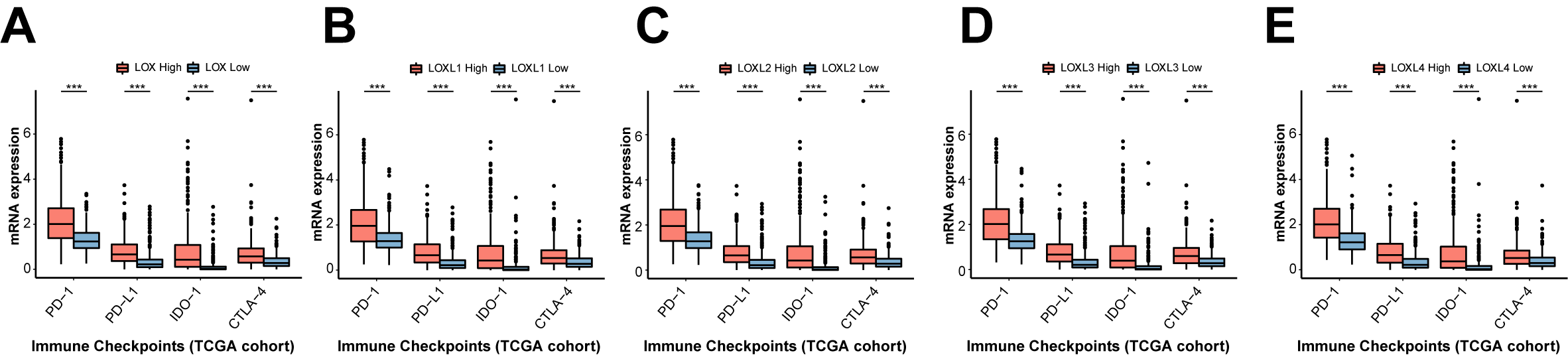

Supplement: Supplementary file 9 [file Image8.TIF]

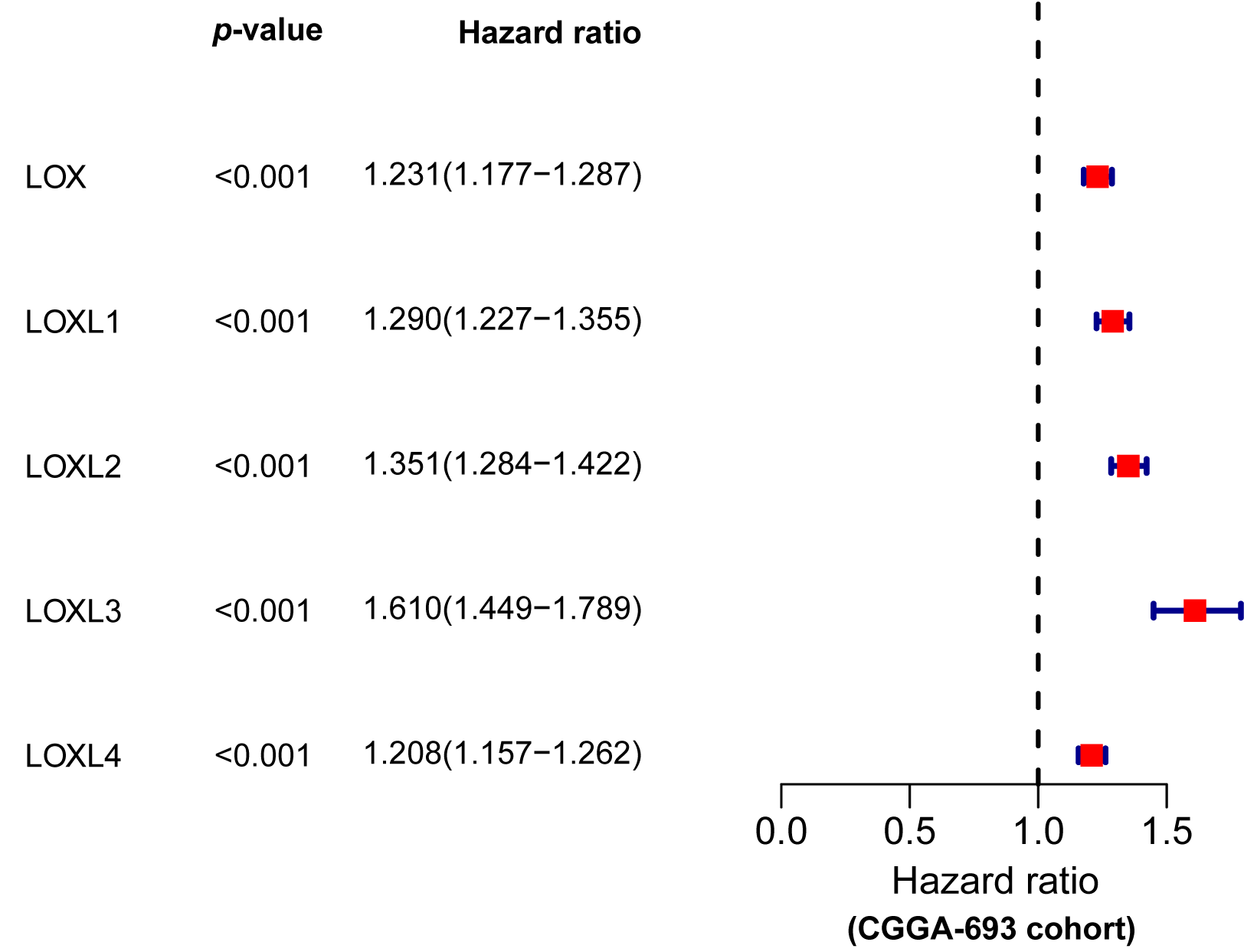

Supplement: Supplementary file 10 [file Image5.TIF]
